# Structures of respiratory syncytial virus G bound to broadly reactive antibodies provide insights into vaccine design

**DOI:** 10.1038/s41598-025-92886-w

**Published:** 2025-03-13

**Authors:** Maria G. Juarez, Sara M. O’Rourke, John V. Dzimianski, Delia Gagnon, Gabriel Penunuri, Vitor H. B. Serrão, Russell B. Corbett-Detig, Lawrence M. Kauvar, Rebecca M. DuBois

**Affiliations:** 1 Department of Molecular, Cell, and Developmental Biology, University of California Santa Cruz, Santa Cruz, CA USA; 2 Department of Biomolecular Engineering, University of California Santa Cruz, Santa Cruz, CA USA; 3 Genomics Institute, University of California Santa Cruz, Santa Cruz, CA USA; 4 Department of Chemistry & Biochemistry, University of California Santa Cruz, Santa Cruz, CA USA; 5 Biomolecular Cryo-Electron Microscopy Facility, University of California Santa Cruz, Santa Cruz, CA USA; 6 Trellis Bioscience, Inc, Redwood City, CA USA

**Keywords:** Immunology, Structural biology, Vaccines, Virology

## Abstract

Respiratory syncytial virus (RSV) is a leading cause of severe lower respiratory tract disease in infants and older adults. The attachment glycoprotein (RSV G) binds to the chemokine receptor CX3CR1 to promote viral entry and modulate host immunity. Antibodies against RSV G are a known correlate of protection. Previously, several broadly reactive, high-affinity anti-RSV G human monoclonal antibodies were isolated from RSV-exposed individuals and were shown to be protective in vitro and in vivo. Here, we determined the structures of three of these antibodies in complex with RSV G and defined distinct conformational epitopes comprised of highly conserved RSV G residues. Binding competition and structural studies demonstrated that this highly conserved region displays two non-overlapping antigenic sites. Analyses of anti-RSV G antibody sequences reveal that antigenic site flexibility may promote the elicitation of diverse antibody germlines. Together, these findings provide a foundation for next-generation RSV prophylactics, and they expand concepts in vaccine design for the elicitation of germline lineage-diverse, broadly reactive, high-affinity antibodies.

**Supplementary Information:**

The online version contains supplementary material available at 10.1038/s41598-025-92886-w.

## Introduction

Lower respiratory infections are the leading cause of infant mortality worldwide with Respiratory Syncytial Virus (RSV) as the primary causative agent^1,2^. RSV induced lower respiratory disease also significantly impacts children under 5, immunocompromised individuals, and the elderly^3–7^. Current FDA approved prophylactic strategies include two monoclonal antibodies, palivizumab (Synagis) and nirsevimab (Beyfortus), and three vaccines, Abrysvo (Pfizer), Arexvy (GSK), and mRESVIA (Moderna), all of which target only one of the two major surface glycoproteins required by RSV for efficient infectivity^8–11^. While these prophylactics are effective at reducing severe lower respiratory symptoms, albeit with efficacy decreasing with age among the elderly, upper respiratory infections are still prevalent and may contribute to RSV shedding and transmission in the community^12–16^.

RSV is a filamentous, enveloped, negative sense, single stranded RNA virus with a 15 kilobase genome coding for 11 proteins^17^. RSV belongs to the *Pneumoviridae *family of viruses which rely on their G and F glycoproteins to mediate attachment and membrane-fusion, respectively, to airway epithelial cells^17,18^. RSV F is a type Ι integral membrane protein and it facilitates fusion between the viral envelope and the host cell plasma membrane by undergoing a series of conformational changes taking it from a pre-fusion to a post-fusion state^18–20^. Antibodies that target the pre-fusion state of RSV F are highly correlated to reduced disease severity. In fact, Nirsevimab, Abryso, Arexvy and mRESVIA were all designed to target antigenic sites Ø and V which are only accessible in this conformation^21–23^. However, known variability localized in these sites highlight the potential for escape from current prophylactics^20,24,25^. This limitation materialized during a phase 3 clinical trial for Suptavumab, an anti-preF neutralizing antibody that failed to meet its efficacy endpoint due to the emergence of an RSV B strain yielding two key mutations in its binding epitope^26,27^. Escape mutations to Nirsevimab have already been identified in circulation, albeit in low abundance^28^. Nevertheless, these escape mutants do not restrict viral fitness in vitro, making it possible for one to emerge as the dominant strain in the future^29^. Escape was also found in 10–30% of subjects (dose-dependent) treated with the small molecule presatovir targeting RSV F^30^.

**Fig. 1:**
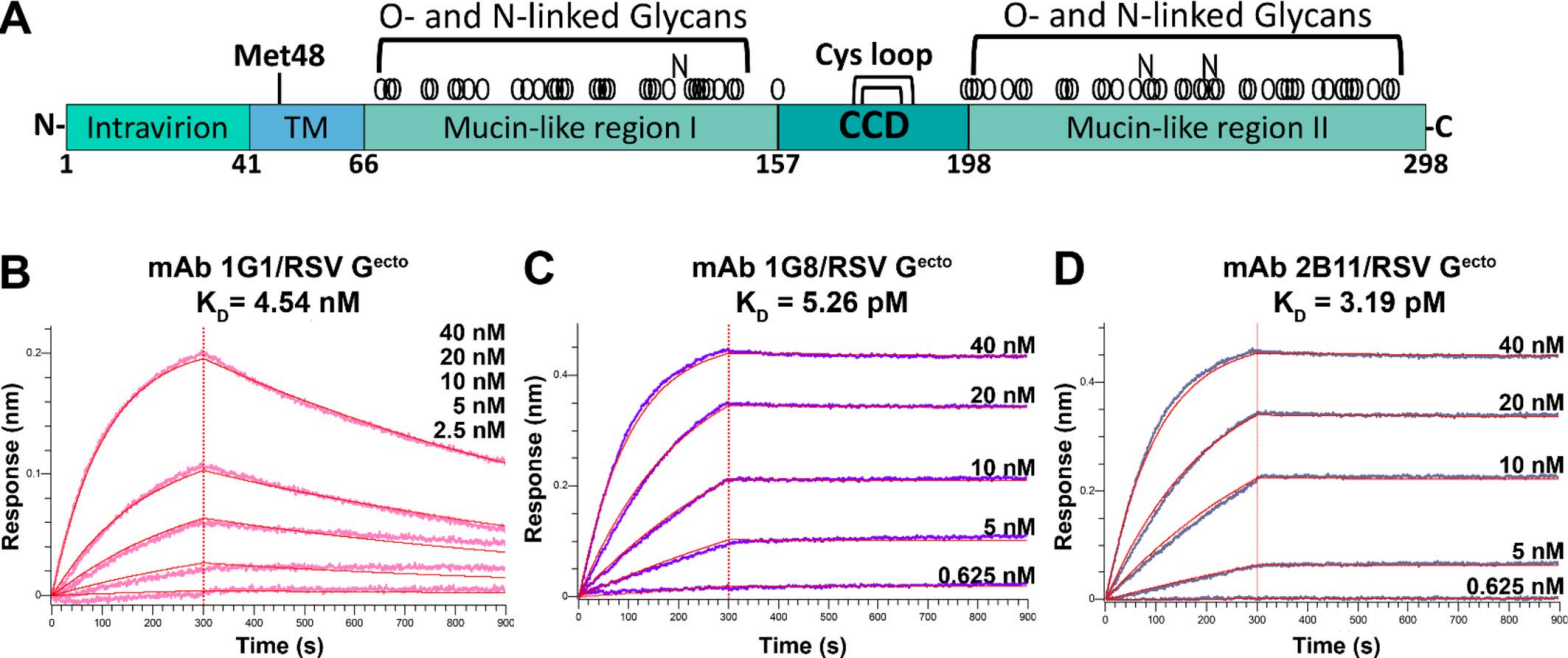
Biolayer interferometry binding studies show that mAbs 1G1, 1G8, and 2B11 bind to RSV G^ecto^ with high affinity. **(A)** Schematic of the RSV G gene. (**B-D)** Biolayer interferometry traces for (**B**) mAb 1G1 (pink traces), (**C**) mAb 1G8 (purple traces), and (**D**) mAb 2B11 (slate gray traces) binding to RSV G^ecto^. Concentrations of RSV G^ecto^ used for each trace are shown. The vertical red line indicates the transition of the biosensors from the association step to the dissociation step. Curve fits using a 1:1 global binding model are colored red. K_D_ values were determined using the average of two technical replicates.

To improve the protective breadth of RSV prophylactics, it is important to consider all correlates of protection which not only includes antibodies targeting pre-fusion RSV F, but those targeting RSV G as well^23,31^. RSV G is a ~ 300 amino acid type II membrane protein. Its extracellular region is composed of two highly-O-glycosylated mucin-like domains flanking a ~ 40 amino acid region known as the central conserved domain (CCD) (Fig. 1A). The CCD contains four cysteines that form two disulfide bonds with 1–4 and 2–3 connectivity. The CCD interacts with CX3CR1 to promote virus attachment and modulate immune responses^32–34^. Antibodies that target RSV G are correlated to reduced disease severity even while being present at < 3% the abundance of those that target the pre-fusion RSV F^23,35^. Mechanistically, anti-RSV G antibodies impede RSV G interaction with the human CX3CR1 receptor and are shown to directly neutralize virus infection in vitro using primary human airway (HAE) and bronchial epithelial cells^32,36,37^. In one study using primary HAE’s, anti-RSV G antibodies targeting the CCD reached up to 92% RSV neutralization; murine mAb 131-2G, a well-studied protective antibody in vivo, reached complete neutralization^37–39^. Anti-RSV G antibodies also mediate opsonization, antibody dependent cellular cytotoxicity (ADCC), and antibody dependent cellular phagocytosis (ADCP) to promote viral clearance^32,33,36,37^. Moreover, anti-RSV G antibodies mitigate the immune modulating activities of RSV G, and using PBMC’s, A549 cells, and a two-chamber transwell in-vitro system, anti-RSV G antibodies were found to restore type Ι and III interferon levels that are normally suppressed by RSV G-CX3CR1 interactions^40^. In vivo, anti-RSV G antibodies work prophylactically and therapeutically to restore a beneficial Th1/Th2 cytokine profile, decrease mucus production, and relieve pulmonary inflammation using both mouse and cotton rat models^36,38,39,41,42^. Notably, the broadly reactive (strain-independent) human monoclonal antibody 3D3 was superior to palivizumab (Synagis), a commercially available anti-RSV F monoclonal antibody, in reducing viral load in mice in both prophylactic and post-infection treatment models^42^. Considering the crucial role of RSV G in viral entry and disease, it is an attractive target for the development of improved prophylactic strategies that could be used alone and/or in combination with those that target RSV F.

In previous studies, five high-resolution crystal structures of human monoclonal antibodies (mAbs) in complex with the RSV G CCD were elucidated^36,43,44^. Unexpectedly, these structures revealed that these mAbs bind to conformational epitopes on the CCD, with additional interactions in the CCD beyond their linear epitopes. However, it is unclear how many conformational epitopes on the CCD exist, if multiple mAbs can bind to the CCD simultaneously, or how mature anti-RSV G human mAb sequences compare to their germline progenitors and to each other. To broaden our understanding of RSV G’s epitope landscape and to understand the basis for elicitation of broadly reactive high-affinity anti-RSV G antibodies from germline lineages, we investigated three broadly reactive high-affinity anti-RSV G human mAbs 1G1, 2B11, and 1G8, which were previously isolated from RSV-exposed individuals^42^. We solved the crystal structures of these antibodies in complex with the CCD and found that each mAb recognizes a unique conformational epitope, distinct from those characterized previously. Moreover, we found that while some mAbs bind to nearly identical CCD amino acids, each antibody makes unique combinations of hydrophobic, electrostatic, and hydrogen bond interactions with either the backbone or sidechain to stabilize contact with the CCD (Fig. 1C). Epitope binning assays and cryoelectron microscopic studies revealed that the CCD can accommodate binding by two broadly reactive monoclonal antibodies simultaneously, supporting the potential for vaccine immunogens that are highly resistant to escape. Finally, mAb sequence analyses of known broadly reactive anti-RSV G human antibodies revealed that they are derived from several different germline lineages, and that they are of modest divergence from their germline genes, suggesting that vaccines will be able to induce such antibodies in a substantial majority of subjects. Altogether, these studies reveal the potential for the RSV G CCD to elicit diverse polyclonal antibody responses that resist virus mutational escape while also providing viral load reduction and unique activity counteracting dysfunctional innate immune responses. This work serves to inform the development of next-generation RSV vaccines and antibody therapeutics.

## Results

### mAbs 1G1, 1G8, and 2B11 bind RSV G with high affinity

Previously, a panel of broadly reactive high-affinity human monoclonal antibodies (mAbs) was isolated from RSV-exposed individuals, and several mAbs were shown to be protective in prophylactic and post-infection mouse models^36,39,41,42,45–47^. Here, we investigated mAbs 1G1, 1G8, and 2B11 from this panel. We selected mAb 1G1 because linear epitope mapping studies had shown no binding to any RSV G linear peptide, suggesting it has a conformational epitope. Additionally, we selected mAbs 1G8 and 2B11 due to their high-affinity binding for RSV. All three mAbs are broadly reactive and bind RSV G from both A and B subtypes^42^. We first generated the recombinant mAbs 1G1, 1G8, and 2B11 and subjected them to kinetics binding experiments with recombinant RSV G ectodomain (RSV G^ecto^) using biolayer interferometry (Fig. 1B, C, D). We found that mAb 1G1 binds RSV G^ecto^ with nanomolar affinity (4.54 nM) while mAb 1G8 and mAb 2B11 bind RSV G^ecto^ with picomolar affinity (5.26 pM and 3.19 pM, respectively) (Fig. 1).

### High-resolution structures of Fabs 1G1, 1G8, and 2B11 bound to RSV G CCD

**Fig. 2:**
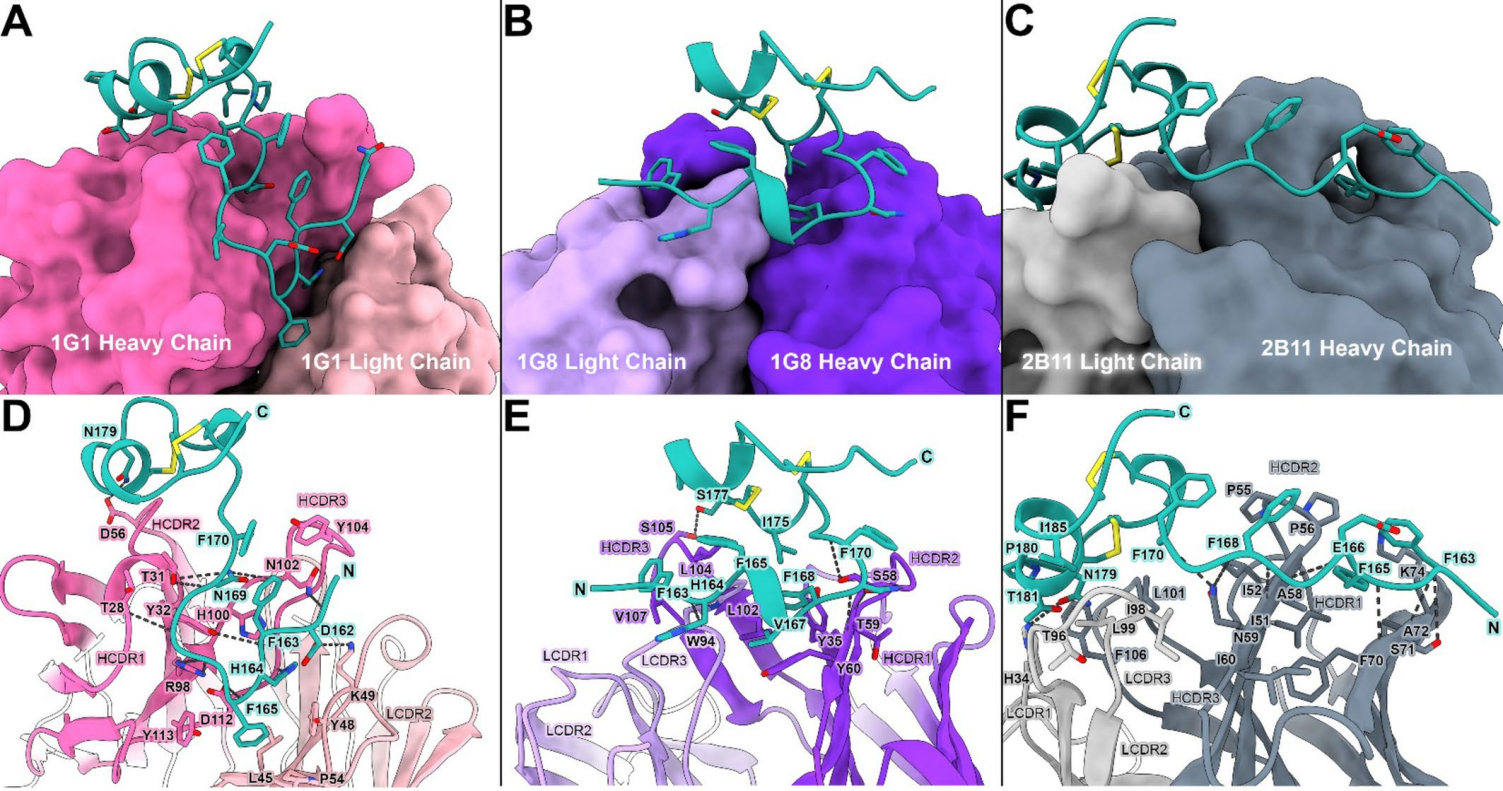
Crystal structures of antibody - RSV G CCD complexes reveal novel conformational epitopes. (**A-C)** Overall surface views of Fab 1G1 (pink), Fab 1G8 (purple), and Fab 2B11 (slate gray) bound to RSV G CCD (cyan). The CCD is shown as stick-and-ribbon view with disulfide bonds colored yellow. (**D-F)** Detailed stick-and-ribbon models depicting the molecular interactions between Fab 1G1, Fab 1G8, and Fab 2B11 and the CCD. Black dotted lines indicate hydrogen bonds. All 3D protein structures in this figure were rendered in ChimeraX version 1.6.1 (https://www.rbvi.ucsf.edu/chimerax)^68^.

To understand the molecular basis for the high-affinities and broad-reactivity of these mAbs, we used X-ray crystallography to determine the high-resolution structures of the 1G1, 1G8, and 2B11 antigen binding fragments (Fabs) in complex with the RSV G CCD (Fig. 2; Table 1). All three mAbs recognize distinct conformational epitopes on the CCD formed by varying conformations of the CCD’s N-terminal region (CCD amino acids 161–171) along with one face of the cysteine loop (CCD amino acids 172–187). These conformational epitopes explain the lack of linear epitope binding for mAb 1G1 and unveil additional epitope amino acids beyond the linear epitopes for mAbs 1G8 and 2B11.

**Table 1:** Data collection and refinement statistics.

	Fab 1G1/RSV G CCD	Fab 1G8/RSV G CCD	Fab 2B11/RSV G CCD
**PDB Code** **Data collection**	9CQA	9CQB	9CQD
Space group	P 21 21 21	P 31 2 1	P 1 21 1
Cell dimensions			
*a*, *b*, *c* (Å)	76.2, 80.75, 175.18	67.39, 67.39, 286.34	74.65, 184.33, 161.23
α, β, γ (°)	90, 90, 90	90, 90, 120	90, 96.87, 90
Resolution (Å)	47.32–1.74 (1.80–1.74)*	286.35–2.50 (2.54–2.50)	80.04–3.10 (3.21–3.10)
*R*_merge_	0.100 (2.346)	0.171 (1.385)	0.412 (1.884)
*I* / σ*I*	14.6 (1.0)	12.7 (1.0)	3.3 (0.4)
CC(1/2)	0.999 (0.457)	0.997 (0.553)	0.984 (0.348)
Completeness (%)	99.1 (94.3)	99.90 (99.62)	99.08 (92.06)
Redundancy	13.2 (9.6)	18.9 (19.6)	13.2 (10.3)
**Refinement**			
Resolution (Å)	47.32–1.74 (1.80–1.74)	58.36–2.5 (2.59–2.50)	79.88–3.1 (3.21–3.10)
No. reflections	110,821 (10422)	27,222 (2663)	78,360 (7747)
*R*_work_ / *R*_free_	0.211/0.225	0.226/0.263	0.292/0.324
No. atoms			
Protein	7451	3578	25,773
Ligand/ion	20	0	0
Water	393	16	0
*B*-factors			
Protein	34.79	54.45	74.50
Ligand/ion	30.0		
Water	39.08	44.81	
R.m.s. deviations			
Bond lengths (Å)	0.008	0.009	0.006
Bond angles (°)	0.97	1.09	0.88
Ramachandran statistics Favored (%) Allowed (%) Outliers (%)	98.56 1.44 0.00	93.07 6.93 0.00	93.55 6.45 0.00

*Values in parentheses are for highest-resolution shell.

The 1G1-CCD complex structure reveals a 1,084-Å^2^interface of which 849-Å^2^is contributed by the heavy chain and 235-Å^2^ is contributed by the light chain (Fig. 2A). Major hydrophobic contacts are mediated by the heavy chain complementarity-determining region (CDR) loops HCDR2 and HCDR3 and light chain CDR loop 2 LCDR2 (Fig. 2D). The N-terminal tail of the CCD is comprised of both polar and non-polar residues that are tucked into a pocket created between the 1G1 heavy chain and light chain. F170 and F163 in the CCD form pi-pi stacking interactions with Y104 in HCDR3 while H164 and F165 in the CCD rest inside a hydrophobic pocket formed by H100 and Y113 in HCDR3 and Y48, P54, and L45 in the LCDR2. A salt bridge is mediated by K49 in LCDR2 interacting with D162 in the CCD. Eleven hydrogen bond interactions, the most seen among the three crystal structures in this study, are predominantly mediated by HCDR2 and HCDR3 with the CCD backbone, a pattern observed previously in the antibody 3G12-CCD interface^44^.

The 1G8-CCD complex structure reveals a 1041-Å^2^interface of which 845-Å^2^is contributed by the heavy chain and 196-Å^2^ is contributed by the light chain (Fig. 2B). The N-terminal tail of the CCD, which includes the known linear epitope amino acids 164–172^42^, wraps around the heavy and light chain, predominantly interacting with HCDR3, HCDR2, and LCDR3. Heavy chain residues V107, L104, L102, Y60, T59, and Y35 and light chain residue W94 form major and minor hydrophobic pockets wherein lie the CCD amino acids F163, F165, V167, F168, and I175 (Fig. 2E). Five out of six hydrogen bond interactions are found between antibody 1G8 and the CCD backbone.

The 2B11-CCD complex structure reveals a 1130-Å^2^interface of which 820-Å^2^is contributed by the heavy chain and 310-Å^2^ is contributed by the light chain (Fig. 2C). This interface is the largest out of the three in this study. Similar to what was observed in the structures with antibodies 1G1 and 1G8, the N-terminal tail of the CCD, which includes the known linear epitope amino acids 162–172^42^, forms intimate interactions with antibody 2B11 using both hydrogen bond and hydrophobic interactions (Fig. 2F). In particular, HCDR2 amino acids I51, I52, P55, P56, A58, I60, and A72 form a hydrophobic interface to interact with the CCD. Interestingly, K74 in the 2B11 heavy chain forms a tunnel with P56 from HCDR2 where the CCD amino acid F165 neatly tucks into. Beyond the linear epitope amino acids, one face of the CCD’s cysteine loop lays flat over 2B11, making prominent interactions with HCDR3, LCDR1, and LCDR3. Specifically, amino acids L101 and F106 in HCDR3 and H34, L99, I98, and T96 in LCDR1 and LCDR3 create a hydrophobic environment that interacts with CCD amino acids P180, T181, and I185 located at the apex of the cysteine loop. In addition, N179 in the CCD forms a hydrogen bond with H34 in LCDR2.

### Antibodies bound to similar RSV G CCD conformations and epitope amino acids utilize distinct molecular interactions

**Fig. 3:**
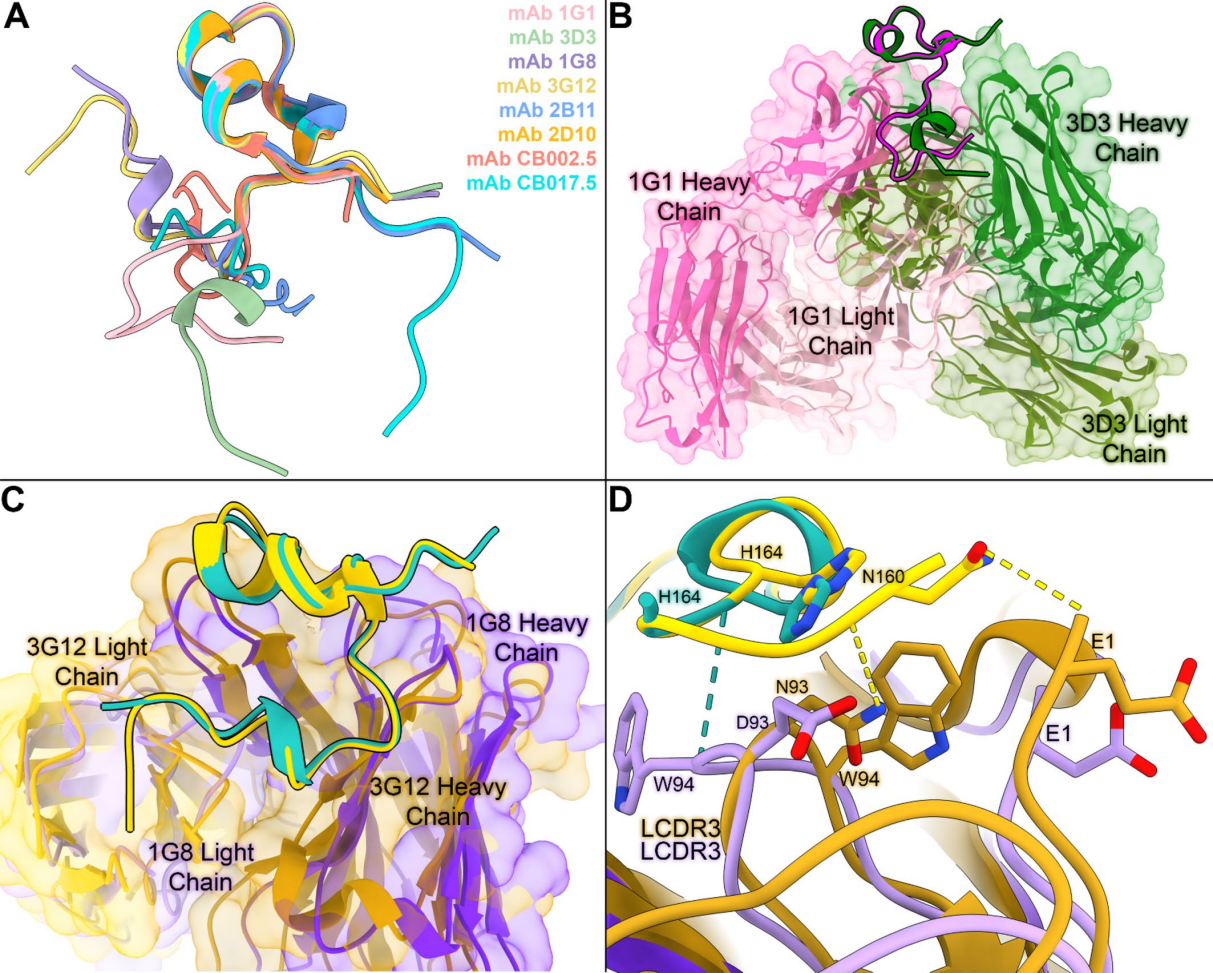
Comparative structural analyses of mAbs bound to similar RSV G CCD conformations reveal distinct interactions. **(A)** Overlay of RSV G CCD structures when bound to antibodies 1G1 (pink, PDB code 9CQA), 3D3 (pale green, PDB code 5WNA), 1G8 (periwinkle, PDB code 9CQB), 3G12 (yellow, PDB code 6UVO), 2B11 (blue, PDB code 9CQD), 2D10 (orange, PDB code 5WN9), CB002.5 (salmon pink, PDB code 6BLI), and CB017.5 (turquoise PDB code 6BLH). (**B)** Overlay of the structures Fab 1G1 (pink surface and ribbon model) and Fab 3D3 (green surface and ribbon model) bound to RSV G CCD (ribbon model in magenta when bound to Fab 1G1 and forest green when bound to Fab 3D3). (**C**,** D)** Overview and zoom-in of the overlay of the structures of Fab 1G8 (purple) and Fab 3G12 (goldenrod) bound to RSV G CCD (ribbon model in sea green when bound to Fab 1G8 and gold when bound to Fab 3G12). In panel C, sea green dotted lines represent hydrogen bonds between Fab 1G8 and RSV G CCD, and gold dotted lines represent hydrogen bonds between Fab 3G12 and RSV G CCD. All 3D protein structures in this figure were rendered in ChimeraX version 1.6.1 (https://www.rbvi.ucsf.edu/chimerax)^68^.

Alignment of the CCDs from the three antibody-CCD structures reported here and the five antibody-CCD structures determined previously reveal that the CCD can adopt several different conformations (Fig. 3A)^36,43,44^. While several of these mAbs bind to the same RSV G amino acids, careful structural analysis reveals that each mAb displays a unique pattern and type of interaction, resulting in a unique epitope. However, two of the structures reported here appear to bind a conformation of CCD that is similar to that in another structure. For example, the RSV G CCD conformation and the epitope amino acids of Fab 1G1 resemble those of Fab 3D3^43^ (Fig. 3B). However, a closer inspection reveals distinct angles of approach and distinct molecular interactions. Specifically, overlayed Fab 1G1-RSV G CCD and Fab 3D3-RSV G CCD structures reveal a 23.9° angle difference in approach by these antibodies for binding to the CCD (Fig. 3B). At the molecular level, Fab 1G1 relies on an extensive hydrogen bond network with the CCD backbone whereas Fab 3D3 relies on sidechain-sidechain interactions^43^ (Fig. 2D). Overall, while antibodies 1G1 and 3D3 bind to nearly identical amino acids on the CCD, their different angles of binding result in different contributions by each CCD amino acid to the epitope (Fig. 3B).

In contrast, Fab 1G8 and Fab 3G12, which are from the same antibody germline (described further below), have similar angles of approach, capture RSV G CCD in a nearly identical conformation, and bind to nearly identical amino acids on the CCD (Fig. 3C). Despite this, molecular nuances distinguish their modes of binding to the CCD. Most notably, W94 from the Fab 1G8 LCDR2 is flipped over and positioned on the opposite side of the RSV G CCD N-terminal tail compared to W94 from the Fab 3G12 LCDR2 (Fig. 3D). This difference allows W94 to create a hydrogen bond with RSV G CCD amino acid H164, an interaction not seen in the Fab 3G12-RSV G CCD interface (Fig. 3D). Also, N93 from the Fab 3G12 LCDR2 acts as a hydrogen bond donor to the carbonyl group of RSV G CCD amino acid N160. At this same position, Fab 1G8 LCDR2 bares E93 which is negatively charged and is unable to act as a hydrogen bond donor.

**Fig. 4:**
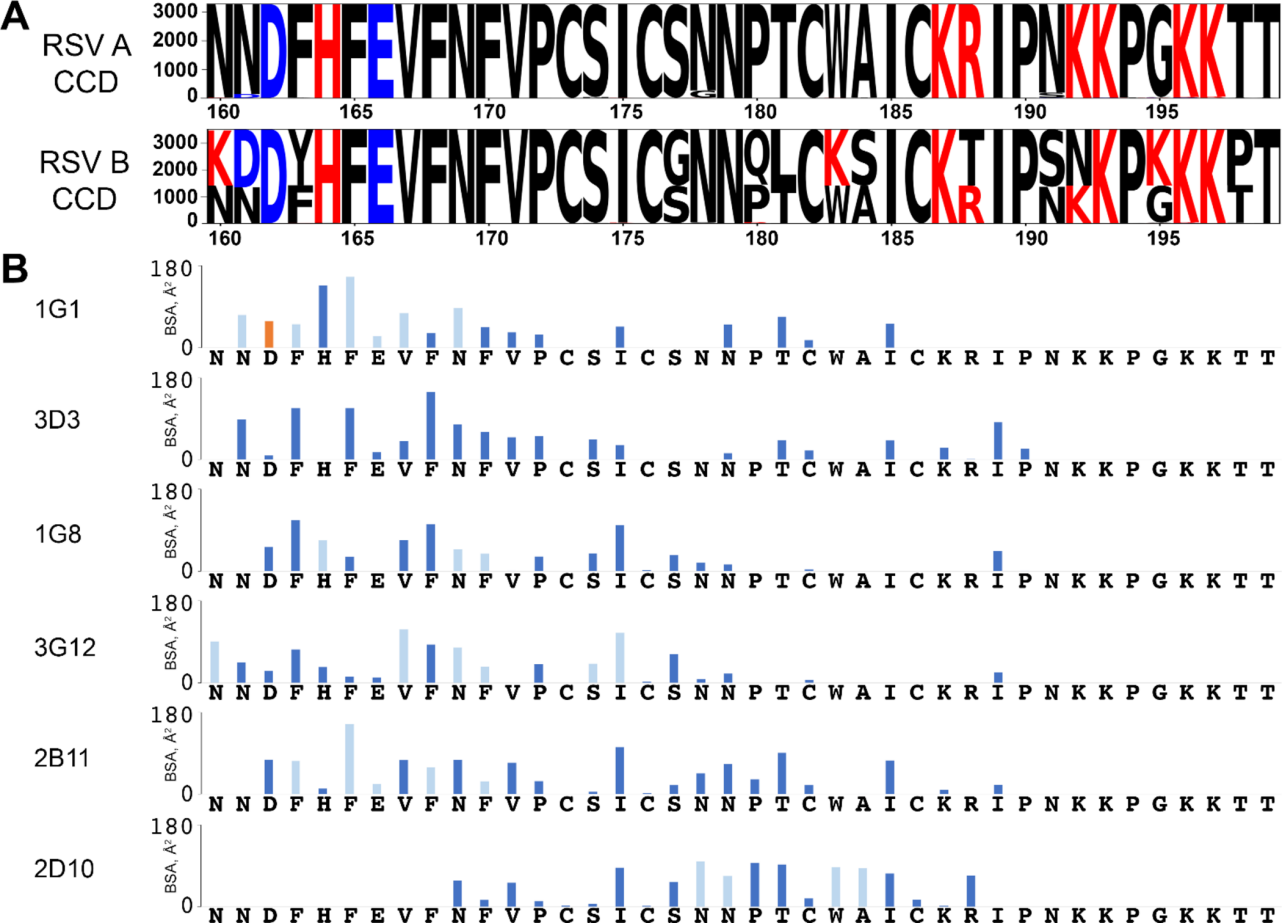
Broadly reactive human mAbs use distinct interactions to bind to RSV G amino acids that are conserved across thousands of RSV genotypes. (**A**) RSV G CCD sequence conservation analysis using the National Center for Biotechnology Information (NCBI) database. (**B)** Buried surface area (BSA) quantitation for each antibody-CCD interface across each CCD amino acid using PDBePISA. Light blue bars indicate antibodies making a hydrogen bond with the CCD peptide backbone. Dark blue bars indicate other hydrophilic or hydrophobic interactions. Orange bars indicate a salt bridge.

Overall, comparative analyses of the epitopes for all six human mAbs from our panel for which we have high-resolution structures is shown in Fig. 4. Analysis of approximately 6000 publicly available RSV G sequences reveals that the CCD is extraordinarily conserved (Fig. 4A). While many of the mAbs bind to the same CCD amino acids, they differ in their mechanism and extent of interaction with each amino acid (Fig. 4B).

### RSV G CCD has two non-overlapping antigenic sites

**Fig. 5:**
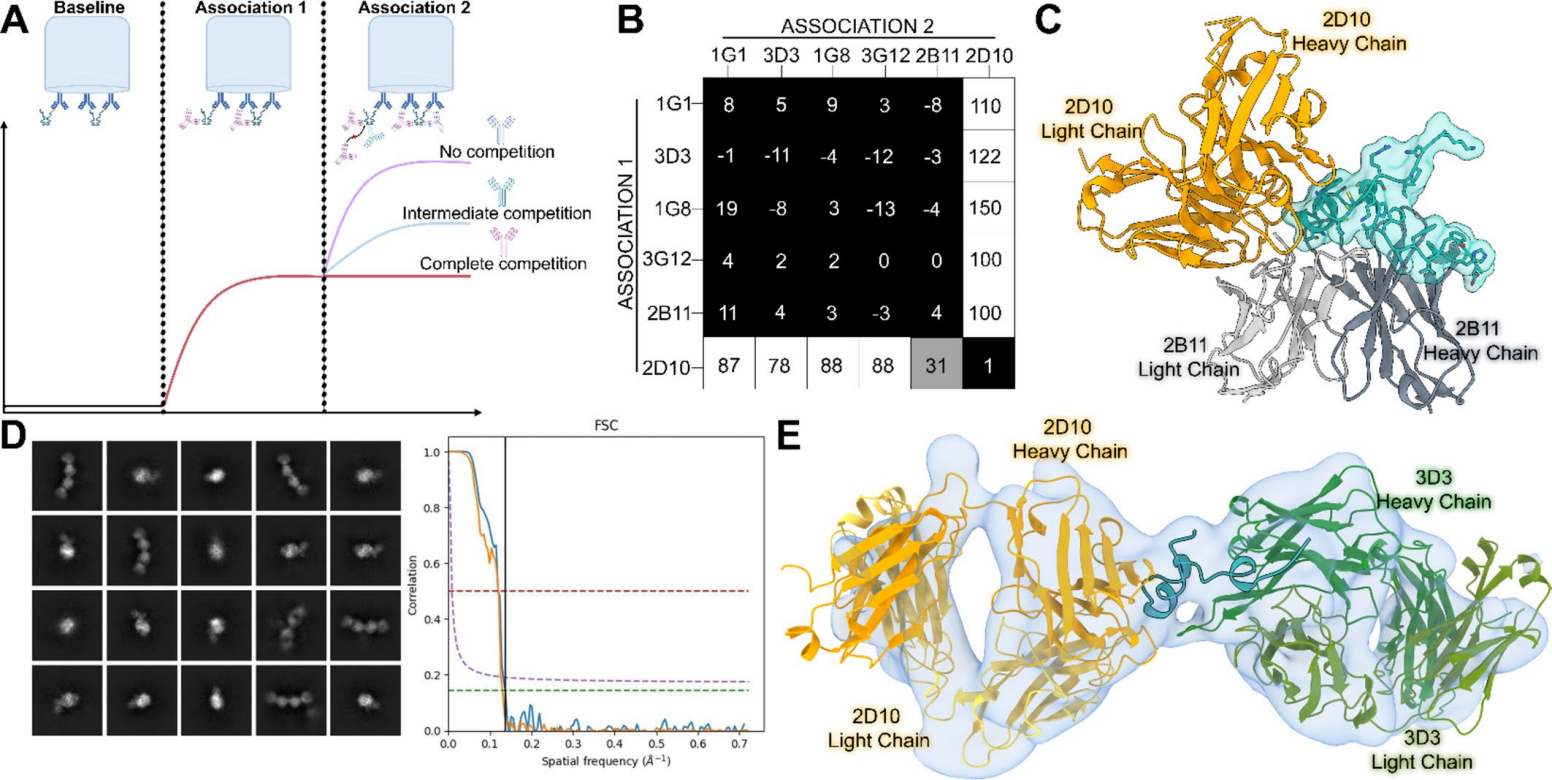
Biolayer interferometry epitope binning and cryo-EM reveal two non-overlapping antigenic sites on the RSV G CCD. (**A**) Graphical description of the epitope binning experimental design using biolayer interferometry. RSV G^ecto^ coated on biosensors (Baseline) is incubated with one mAb to saturation (Association 1) and then incubated with a second mAb to evaluate binding (Association 2). (**B**) Epitope binning data showing the extent of competition between the mAbs in the Association 1 and 2 steps. Higher numbers (white boxes) indicate strong reactivity of the mAb in Association 2 in the presence of the mAb in Association 1 (no competition) and lower numbers (black boxes) indicate complete competition. Data represents the average of two technical replicates. (**C)** Overlay of the crystal structures Fab 2B11 (slate gray) and antibody scFv 2D10 (orange) bound to RSV G CCD (cyan). (**D**) Representative 2D classes and Fourier shell correlation (FSC) graph. (**E)** Approximately 6.9 Å-resolution cryo-EM map and model of Fab 2D10 and Fab 3D3 bound to RSV G^ecto^. The model was generated using the overlaid crystal structures of the scFv 2D10 - RSV G CCD and Fab 3D3 - RSV G CCD complexes which was then fit into the map using ChimeraX Isolde^68,76^. All 3D protein structures in this figure were rendered in ChimeraX version 1.6.1 (https://www.rbvi.ucsf.edu/chimerax)^68^.

To determine if the RSV G CCD, which is only ~ 40 amino acids, displays antigenic sites accessible by more than one antibody at the same time, we conducted an epitope binning assay with the six human mAbs from our panel for which we have structural information. Biolayer interferometry biosensors coated with recombinant RSV G glycoprotein were used to evaluate competitive binding by each mAb against the other, allowing the generation of a competitive binding matrix (Fig. 5A, B). Antibodies 1G1, 3D3, 1G8, 3G12, and 2B11 all competed against each other for binding to RSV G, confirming that they share the same antigenic site, termed γ1, consistent with their structures and overlapping epitopes (Fig. 5B). In contrast, antibody 2D10 was the only antibody in our panel that did not compete for binding to RSV G, supporting that it binds to a distinct antigenic site, termed γ2. Interestingly, antibody 2B11 appears to compete intermediately with antibody 2D10 for binding to RSV G when it is introduced in the second association step. This can be explained through its slight overlap with antibody 2D10 when viewing the overlaid structures (Fig. 5C). No competition was observed when antibody 2B11 was introduced in the first association step, likely due to the higher affinity of antibody 2D10 (K_D_ < 1pM) compared to antibody 2B11 (K_D_= 4.5 nM)^44^.

Taking structural data and epitope binning data together, we propose antigenic site γ1 at residues N160-P172 and antigenic site γ2 at residues C173-R188. To further support this antigenic model, we incubated non-competing Fabs 2D10 and 3D3 with recombinant RSV G glycoprotein and used this sample to collect cryoelectron microscopy (cryo-EM) data (Fig. 5D, Supplementary Fig. 1). A ~ 6.9 Å-resolution 3D volume shows the relative shape of two Fabs bound at one focal point (RSV G CCD)(Fig. 5E). The RSV G mucin-like domains are highly flexible and were not seen in either our raw or processed data. Nevertheless, we created an overlay of the crystal structures of Fabs 2B11 and 3D3 bound to RSV G CCD and modeled this into our 3D volume (Fig. 5E). These data serve to corroborate the existence of two antigenic sites and is the first time that RSV G bound to two antibodies simultaneously has been visualized.

### Anti-RSV G antibodies have modest divergence from genomic precursors

B-cell precursors undergo a process called VDJ recombination where selected variable (V), diversity (D), and joining (J) genes are rearranged as the B-cell matures and migrates to secondary lymph nodes. These naïve B-cells reside in the secondary lymph organs until they are activated, at which point, they are instructed to enter the light zone of the germinal center and undergo affinity maturation, the process of creating nucleotide substitutions or insertions in CDR1 and CDR2 (coded by the V gene) and CDR3 (coded by the D and J gene in the heavy chain and the V and J genes in the light chain). To understand the genetic basis for the elicitation of broadly reactive high-affinity anti-RSV G antibodies, we analyzed variable heavy chain (VH) and variable light chain (VL) germline gene usage for each antibody from our panel of 13 mAbs and nine other anti-RSV G human mAbs with publicly available sequences^36,42,48^. We found 11 distinct VH gene precursors and 12 distinct VL gene precursors across these twenty-two anti-RSV G mAbs. Surprisingly, these mAbs show relatively high sequence identity to their germline genes (77.6–95.9% in VH and 81.1–97.9% in VL) indicating moderate levels of somatic hypermutation (Table 2). Additionally, we find HCDR3 lengths to be 8–18 amino acids long. Most antibodies use a unique set of heavy and light chains except for antibodies 1G8, 3G12, and 1A5 (IGHV4-39*01 and IGKV3-15*01) along with antibodies AT50 and AT51 (IGHV1-18*01 and IGKV1-9*01).

**Table 2.**
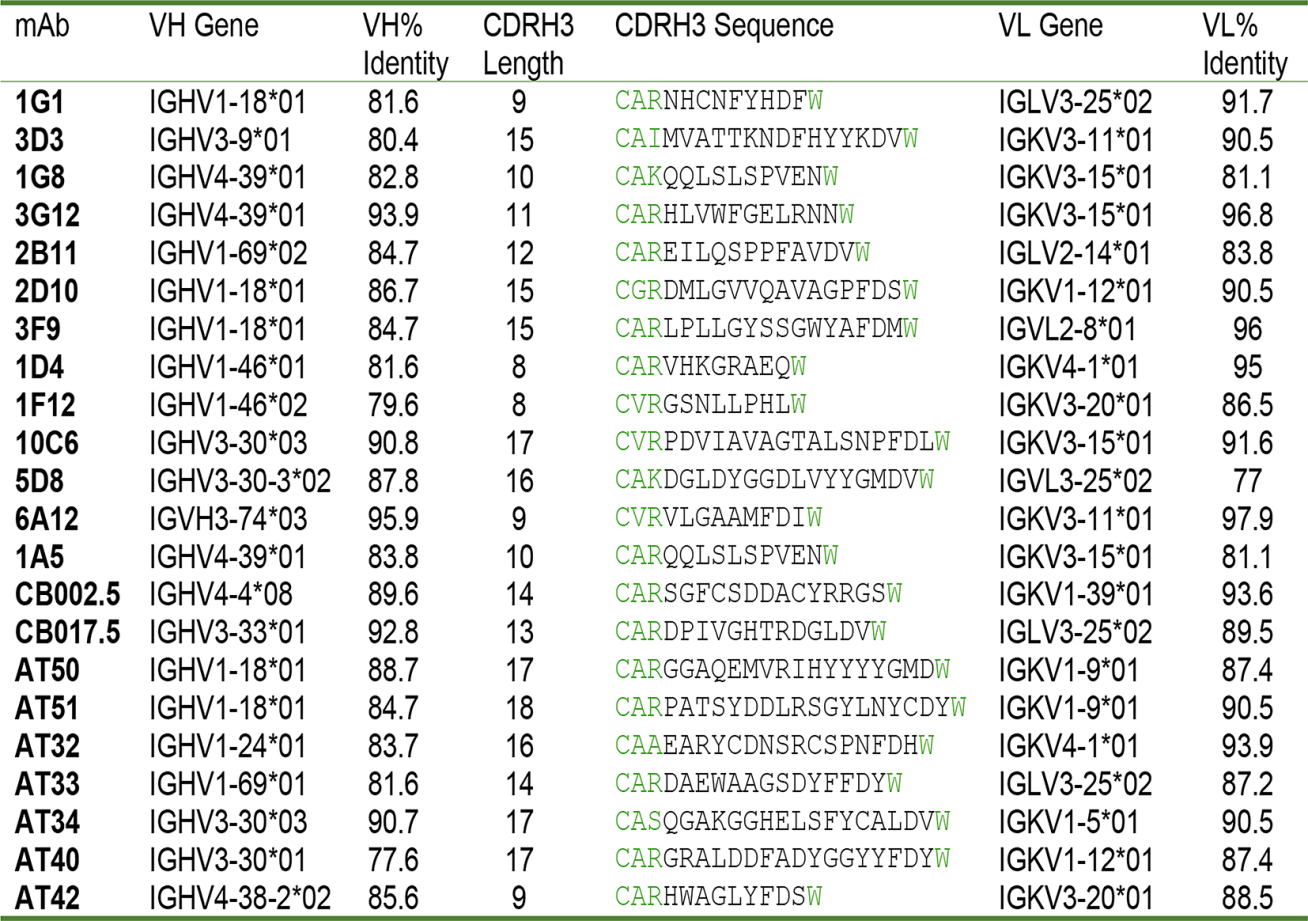
Germline gene sequence identity.

Green letters indicate the framework region flanking CDRH3 (Kabat or Chothia numbering scheme).

## Discussion

Here, we defined three novel highly conserved conformational epitopes on the RSV G CCD using X-ray crystallography, and we visualized two non-competing antigenic sites (γ1 and γ2) using cryo-EM. We find that the CCD, while only 40 amino acids in length, can adopt multiple conformations that are recognized by diverse antibody paratopes. For mAb 1G1, a linear epitope could not be mapped^42^, and now we understand that its reactivity with RSV G can be explained via a unique conformational epitope. Of note, mAb 1G1’s extensive hydrogen bond network with the RSV G CCD N-terminal tail is largely composed of peptide backbone amino and carbonyl groups, leaving open the possibility that it may be more resistant to mutations in the CCD compared to other antibodies, although its affinity is not as strong as other antibodies in the panel. For mAbs 1G8 and 2B11, we find that these antibodies also recognize conformational epitopes, which include amino acids beyond their linear epitopes^42^. Epitope binning studies reveal that five out of the six mAbs investigated are able to compete with each other for binding to an overlapping site, termed antigenic site γ1, whereas mAb 2D10 binds to a distinct epitope, termed antigenic site γ2. Interestingly, mAb 2B11 showed partial competition with mAb 2D10, which can be explained by a partial overlap of their epitopes on opposite faces of the CCD’s cysteine loop.

It is well known that extensive genomic divergence via somatic hypermutation (SHM) is a common feature of broadly-neutralizing high-affinity antibodies, such as those targeting HIV and influenza virus^49–52^. The broadly neutralizing anti-HIV-1 antibody VRC01 variable heavy chain has a sequence identity of 58.2% to its germline gene IGVH1-2*02^49^. In a similar vein, broadly-neutralizing anti-influenza antibody C05 possesses a 24 amino acid long HCDR3 which it utilizes to insert into the HA receptor binding site^50^. In contrast, broadly-neutralizing high-affinity antibodies targeting RSV G diverge modestly from their germline sequences, with the lowest sequence identity being 77.6% and with the longest HCDR3 at 18 amino acids in length across 22 human monoclonal antibodies. While the VRC01 antibody has an HCDR3 length of only 12 amino acids, it relies more heavily on its HCDR2 to form the highest surface area interface with the HIV gp120 protein and takes advantage of its LCDR1 to make important contacts with an HIV gp120 N-linked glycan^49^. Similarly, Fab 2B11 and Fab 1G8 appear to rely more heavily on their HCDR2 and light chain residues and possess only 12 and 10 amino acid long HCDR3s, respectively. However, antibody 1G1 uses its CDRH3 to a higher degree, making its length an important factor when we consider how divergent it might be from its germline gene. It is important to consider the role that RSV G’s flexibility has in the generation of the high affinities (K_D_s from nM to pM) observed in these antibodies. It is possible that the CCD’s flexibility allows it to conform to bind to germline antibodies with an already relatively high affinity, circumventing their need to undergo high SHM to achieve high affinity, a feature that could make the CCD an excellent target for vaccines.

Another feature of some broadly-neutralizing antibodies is that they can be restricted in their antibody germline usage. For example, the highly potent anti-influenza HA mAb CR6261 belongs to a panel of thirteen antibodies, all of which use the same VH gene (IGHV1-69), making it evident that mAb diversity is severely restricted for this antigenic site. In stark contrast, our analysis of 22 anti-RSV G antibodies use 11 unique VH genes in combination with 12 unique VL genes. Elicitation of diverse anti-RSV G antibodies is likely the result of the CCD’s display of many different conformational epitopes via highly flexible N- and C-termini.

In a larger sense, these studies support a key concept for vaccine design: preservation of antigen flexibility. A current strategy in vaccine design is antigen stabilization, primarily in virus membrane-fusion glycoproteins to stabilize them in their pre-fusion conformations. Addition of proline substitutions, disulfide bonds, and cavity-filling mutations have served as the main strategies for antigen stabilization. However, we propose that overstabilization and reduction of antigenic site flexibility could be detrimental, as it may limit polyclonal antibody breadth, which in turn may allow virus escape. Here, we provide evidence that the flexibility of RSV G promotes high-affinity antibody binding and the stimulation of diverse antibody lineages. Moreover, when flexible antigenic sites contain conserved amino acids, as in the case of the RSV G CCD, virus escape may be even more difficult.

Altogether, our studies reveal the RSV G CCD as an ideal vaccine target due to its high sequence conservation and its high flexibility that promotes the elicitation of diverse, high-affinity, and broadly-neutralizing antibodies. These features are especially notable for such a small antigenic target of ~ 40 amino acids. Moreover, the existence of two non-competing antigenic sites, γ1 and γ2, on the CCD further supports its potential to elicit antibody responses that resist virus escape. However, we acknowledge that there has been little selective pressure for the RSV G CCD to mutate due to its naturally low immunogenicity. Thus, it is unclear if selective pressure from a CCD-focused vaccine would result in RSV mutational escape, and future studies focused on this area would be informative. A limitation to performing escape studies in vitro is that anti-G antibodies are most readily assessed for RSV-neutralization only in primary airway epithelial cells, making such studies technically challenging^32,36,37,42^. Overall, this study broadens our understanding of the RSV G epitope landscape and serves as a blueprint for structure-guided vaccine design and therapeutic antibody strategies.

## Methods

### Expression and purification of mAbs

Synthetic genes encoding the heavy-chain or light-chain variable regions of the human mAbs 1G1, 3D3, 1G8, 2B11, 2D10, and 3G12 were cloned into the pCMVR VRC01 light and heavy chain human IgG1 antibody vectors obtained through the NIH HIV Reagent Program, Division of AIDS, NIH: (ARP 12036, ARP-12035) contributed by John Mascola, in place of the variable regions of antibody VRC01. The plasmids were verified by Sanger sequencing. Recombinant mAbs were expressed by electroporation of suspension adapted Chinese Hamster Ovary Cells (CHO-S Freestyle) (Thermo Fisher, Life Technologies) with mAb heavy and light chain plasmids. Prior to transfection, CHO-S cells were maintained at a density of 0.3–2.0 million cells/mL in CD-CHO medium supplemented with 8 mM GlutaMax, 100 µM hypoxanthine and 16 µM thymidine in shake flask cultures at 37 °C, 8% CO_2_, 85% humidity, rotating at 135 rpm in an ISF1-X shaker incubator (Kuhner). For protein production, cells were transfected using an STX electroporation system (MaxCyte Inc.) according to the manufacturer’s protocol using endotoxin free DNA. Following transfection, cells were maintained at a density of 10 million cells /mL in CD OptiCHO supplemented with 0.1% pluronic acid, 2 mM GlutaMax, 100 µM hypoxanthine and 16 µM thymidine. At 24 h post electroporation, protein expression was enhanced by adding sodium butyrate (1 mM final concentration) and lowering the temperature to 32 °C. Cultures were fed daily with CHO Growth A feed which comprises 0.5% Yeastolate (BD, Franklin Lakes, NJ, US), 2.5% CHO-CD Efficient Feed A, and 0.25 mM GlutaMax, 2 g/L Glucose (Sigma-Aldrich) and harvested when cell viability dropped to below 50% (usually following 8–14 days in culture). All media and supplements were purchased from Thermo Fisher, Life Technologies, unless stated otherwise. Secreted mAbs were purified from 0.2 micron filtered conditioned media by affinity chromatography using a HiTrap Protein G column (Cytiva) in PBS buffer [pH7.4]. Bound antibody was eluted with 0.1 M glycine•HCl pH 2.8 and immediately neutralized with 10 µL/mL 1 M Tris-Cl pH 9.0. Purified mAbs were verified by Coomassie stained SDS-PAGE with both reducing and non-reducing loading buffer, dialyzed into phosphate-buffered saline (PBS) pH 7.4 and flash frozen for long term storage.

### Expression and purification of Fabs

Fabs 3D3 and 2D10 were generated by incubation of 4.0 mg full length mAb with 62.5 µg equilibrated immobilized papain at 37 °C overnight, followed by removal of the Fc fragment with protein A using a Pierce™ Fab Preparation Kit (Thermo Fisher, Life Technologies). Fabs were further purified by Superdex 200 size-exclusion chromatography in 10 mM Tris-HCl pH 8.0 and 150 mM NaCl. Purified Fabs were dialyzed into PBS pH 7.4, and flash frozen for long term storage. For the generation of Fabs 1G1, 2B11, and 1G8, Fab expression plasmids encoding the heavy-chain variable and constant region 1 in frame with a C-terminal Twin-StrepTactin-Tag were cloned. Sanger sequencing was used to verify these plasmids. Fabs were expressed in an identical manner to mAbs, in CHO-S culture. Secreted Fabs were affinity purified from 0.2 micron filtered conditioned media adjusted to 50 mM Tris-Cl pH 8.0, 1mM EDTA, supplemented with 27 mg/L Biolock (IBA life sciences), using a StrepTrap HP column (Cytiva) equilibrated in 50 mM Tris-Cl pH 8.0, 150 mM NaCl and 1 mM EDTA wash buffer. After column washing, Fabs were eluted with wash buffer containing 2.5 mM d-Desthiobiotin. Purified Fabs were verified by Coomassie stained SDS-PAGE with both reducing and non-reducing loading buffer and dialyzed into PBS pH 7.4 and flash frozen for long term storage.

### Antibody germline gene sequence identity analysis

Using the IgBlast tool on the National Center for Biotechnology Information (NCBI) website, we input each mAb heavy chain or light chain protein sequence, selecting the top hit for each variable (V) gene and allele to include in our table. Additionally, we determined the CDRH3 sequences using the Kabat or Chothia numbering scheme as a guide^53^.

### RSV G gene sequence analysis

We performed a blastp search with the RSV A sequence (UniProtKB/Swiss-Prot: P03423.1) and the RSV B sequence (GenBank: AGG39486.1)^54,55^. We kept the top 5000 hits ranked by e-value for each sequence and used a custom python script to collect the amino acid regions encompassing the CCD region for these top hits. We then performed a multiple sequence alignment for both the RSV A and RSV B blastp hits using Clustal Omega^56^. The alignments were inspected visually and trimmed with trimAl using the ‘automated1’ flag^57^. The alignment files were then used to create the sequence logos using the python package logomaker within a custom python script^58^.

### Expression and purification of RSV Gecto proteins

A synthetic gene encoding the RSV strain A2 (RSV/A2) G glycoprotein ectodomain (RSV G^ecto^) spanning amino acids 64–298 (UniProtKB entry P03423) was codon optimized and cloned into a pcDNA3.1 derivative plasmid in frame with a N-terminal CCR5 secretion signal sequence and a C-terminal 6xHistidine-Tag followed by a Twin-StrepTactin-Tag. Sanger sequencing was used to verify this plasmid. All recombinant RSV G^ecto^ proteins were produced from stable G418 selected pools generated by transfection followed by selection in 0.8 mg/mL G418 (Thermo-Fisher Scientific). For protein expression, a pool of the G418 resistant cells was grown to a density of 5 million cells/mL in BalanCD CHO Growth A - Irvine Scientific (FujiFilm) supplemented with 2 mM GlutaMax, 100 µM hypoxanthine and 16 µM thymidine and 0.1% pluronic in standard shake flask culture. The temperature was then dropped to 32 °C, and sodium butyrate added to a final concentration of 1 mM. The cultures were supplemented as described above for transient CHO-S expression, with the exception that they were batch-fed on a 3 day regime with 10% v/v CHO A Feed. Secreted RSV G^ecto^ was purified from CHO media adjusted to pH 8.0 by the addition of Tris-Cl buffer to a final concentration of 50 mM, supplemented with 1mM EDTA and Biolock (IBA life sciences) at 27 mg/L. Protein was recovered by affinity purification using a StrepTrap HP column (Cytiva) equilibrated in 50 mM Tris-Cl pH 8.0, 150 mM NaCl and 1 mM EDTA wash buffer. After washing, RSV G^ecto^ was eluted with wash buffer containing 2.5 mM d-Desthiobiotin. CHO-S derived RSV G^ecto^ was further purified by size exclusion chromatography on a Sepharose 6B column (Cytiva) equilibrated with 50 mM Tris-HCl pH 8.0, 300 mM NaCl, and 1 mM EDTA. Fractions were analyzed by Coomassie stained SDS-PAGE, pooled and verified by SDS-PAGE in both reducing and non-reducing loading buffer before dialysis into PBS pH 7.4 and flash frozen for long term storage (Supplementary Fig. 2). A biotinylated version of RSV G^ecto^ was expressed in an identical manner from a plasmid encoding RSV G^ecto^ with a C-terminal 10XHis tag followed by an AviTag sequence. Purification was accomplished on a Ni Sepharose Hi Trap excel column (Cytiva) equilibrated with 20 mM sodium phosphate pH 7.4 and 0.5 M NaCl (wash buffer). Conditioned CHO media was adjusted to pH 7.4 and 0.5 M NaCl prior to column loading. Wash buffer with 20 mM imidazole was used to strip low affinity contaminants and a 20 mM to 500 mM imidazole gradient was used to elute bound protein. Eluted fractions were analyzed by SDS-PAGE prior to pooling. RSV G^ecto^AviTag was then dialyzed into PBS pH 7.4 and biotinylated using GST-BirA ligase as described by Fairhead and Howath^59^. RSV G^ecto^ with GalNAc-restricted O-glycosylation was generated specifically for structural studies by expression of RSV G^ecto^in SimpleCell (Cosmc-KO) GALNT3 + suspension-adapted CHO K1 cells, a gift from H. Clausen at the University of Denmark^60^. Stable RSV G^ecto^ SC ZFN192 GALNT3 cells were generated by electroporation followed by selection in 0.8 mg/mL G418. For protein expression, a pool of the G418 resistant cells was grown to a density of 5 million cells/mL in BalanCD CHO Growth A - Irvine Scientific (FujiFilm) supplemented with 2 mM GlutaMax, 100 µM hypoxanthine and 16 µM thymidine and 0.1% pluronic in standard shake flask culture. The temperature was then dropped to 32 °C, and sodium butyrate added to a final concentration of 1 mM. The cultures were supplemented as described above for transient CHO-S expression, expecting that cultures were batch fed on a 3-day regime with 10% v/v CHO A Feed. Cultures were harvested when cell viability dropped < 50% by trypan blue exclusion, and protein was purified on a StrepTactin Sepharose HP column (as previously described for CHO-S produced RSV G^ecto^) followed by size exclusion chromatography on a Superose 6 increase 10/300 column (Cytiva) in 50 mM Tris-Cl pH 8.0, 300 mM NaCl, and 1mM EDTA and verified by Coomassie stained SDS-PAGE and Blue Native PAGE. The protein was then dialyzed into TBS (50 mM Tris-Cl, pH 7.5 150 mM NaCl) before flash freezing (Figure S5).

### Binding kinetics

Binding kinetics were assessed using biolayer interferometry on an Octet Red384 instrument. Anti-Human Fc-Capture AHC biosensors (Sartorius, REF 18 − 0015) were equilibrated in Octet Kinetics Buffer (Sartorius, #18–1105, formulation: 0.1% BSA, 0.02% Tween20, PBS pH 7.4, and Kathon) diluted to 1X using PBS pH 7.4. Biosensors were then dipped into wells containing kinetics buffer for 60 s to record a baseline measurement. Next, biosensors were loaded with either mAb 1G1, mAb 1G8, or mAb 2B11 at a concentration of 5 µg/ml for 240 s followed by another baseline step in kinetics buffer for 120 s. Loaded biosensors were then dipped into wells containing two-fold serial dilutions of purified RSV G^ecto^ for 300 s with the highest concentration being 40 nM and the lowest being 0.625 nM. The last biosensor was dipped into kinetics buffer which served as a control and to subtract non-specific upward or downward drift from our measurements. A dissociation step was measured for 600 s after moving biosensors into wells containing kinetics buffer. All steps were done with a shakespeed of 1000 rpm and at 24 °C. Two technical replicates were done per antibody. Binding dissociation constants (K_D_s) were calculated using the Data Analysis HT software version 11.1 with a 1:1 binding model and curves fitted globally per assay.

### Epitope binning

Epitope binning was performed using biolayer interferometry on an Octet Red384 instrument and following suggestions described by Nagashima and Mousa^61^. Anti-penta histidine HIS1K biosensors (Sartorius, REF 18–0038) were equilibrated in Kinetics Buffer. Biosensors were then dipped into wells containing kinetics buffer for 60 s to record a baseline measurement. Next, biosensors were loaded with RSV G^ecto^ at a concentration of 4 µg/ml for 150 s followed by another baseline step for 60 s. Loaded biosensors were then dipped into wells containing either mAb 1G1, mAb 2D10, mAb 3G12, mAb 3D3, mAb 2B11, or mAb 1G8 at concentration of 100 µg/ml for 300 s in the first association step. The second association step was done by dipping into either mAb 1G1, mAb 2D10, mAb 3G12, mAb 3D3, mAb 2B11, or mAb 1G8 at a concentration of 100 µg/ml for 300 s so that each antibody was tested for competition against all other antibodies in addition to itself. Antibodies in the first association step were also tested in the second association step to test antibodies in both directions and observe the effect of steric hindrance which might occur after the first association step. A “no load” empty biosensor was used to test for non-specific antibody binding to the biosensor itself, and a 0 µg/ml concentration of antibody in the first association step was as in another control to verify complete saturation in the second association step. Two technical replicates were done per antibody and per order of association. mAb 2B11 was assessed using anti-streptavidin SA biosensors (Sartorius, REF 18 − 0009) and biotinylated RSV G^ecto^ due to its apparent binding to anti-penta histidine HIS1K biosensors in the “no load” control assay despite the absence of a His-tag or StrepTactin-tag. All assays were done with a shakespeed of 400 rpm at 24 °C. To generate a value for competition between two mAbs, the signal of mAb B (second association step) in the presence of mAb A (first association step) was divided by the saturated signal of mAb A and then multiplied by 100 for a percentage value.

### Expression and purification of RSV G CCD

A synthetic gene encoding the RSV strain A2 (RSV/A2) G protein amino acids 157 to 197 (UniProtKB entry P03423) (RSV G CCD) was codon optimized for E. *coli* and cloned into pRSFDuet-1 in frame with an N-terminal methionine and a C-terminal 6xHistidine-Tag. Sanger sequencing was used to verify this plasmid. Two methods were used to express and purify recombinant RSV G CCD as a result of optimization. Method 1: Recombinant RSV G CCD was expressed in T7Express *E. coli* cells (New England Biolabs, REF C2566H) cells overnight at 18 °C. Cells were lysed by ultrasonication in wash buffer (20 mM Tris-Cl pH 8.0, 25 mM imidazole, 150 mM NaCl) containing 1 mM MgCl_2_, protease inhibitors, and benzonase. *E. coli* lysates were clarified by centrifugation and filtration using a 0.22 μm vacuum filter. RSV G CCD was purified from clarified lysates by affinity chromatography using a HiTrap TALON Crude column (GE Healthcare, REF 28–9537−66) and washed with a wash buffer containing 6 M urea. Protein was eluted in wash buffer containing 500 mM imidazole. Method 2: Recombinant RSV G CCD was expressed in SHuffle T7Express Competent *E. coli* cells (New England Biolabs, REF C3026J) overnight at 18 °C. Cells were lysed by ultrasonication in wash buffer (20 mM Tris-Cl pH 8.0, 25 mM imidazole, 300 mM NaCl) containing 1mM MgCl_2_, protease inhibitors, and benzonase. *E. coli* lysates were clarified by centrifugation and filtration using a 0.22 μm vacuum filter. Clarified lysates were mixed with 1 ml of packed HisPur Cobalt Resin (Thermo Scientific, REF 89965) and rotated for 1 h at 4 C. Recombinant RSV G CCD was eluted on a centrifuge column (Pierce, REF 89898) using wash buffer containing 500 mM imidazole and verified on Coomassie stained SDS-PAGE with both reducing and non-reducing loading buffer.

### Production and structure determination of fab 1G1-RSV G CCD complex

A 5-molar excess of recombinant RSV G CCD at 0.1 mg/ml was mixed with Fab 1G1 at 1.5 mg/ml and concentrated to 2.8 mg/ml. This sample was purified on a Superdex75 10/300 size exclusion chromatography (SEC) column with 50 mM Tris-HCl (pH 8) and 150 mM NaCl. SEC-purified Fab 1G1-RSV G CCD complex was verified by Coomassie stained SDS-PAGE and was concentrated to 5 mg/ml. Crystals were grown by hanging drop vapor diffusion at 22 °C with well solution containing 0.1 M Bis-Tris pH 6.5, 0.2 M Ammonium Sulfate, and 24.5% PEG 3350. Crystals were dipped into a cryoprotectant (well solution containing 25% PEG400) and flash frozen in liquid nitrogen. Frozen crystals were shipped to the Advanced Photon Source (APS) and diffraction data was collected at beamline 23-ID-B using an x-ray wavelength of 1.03 Å (12 keV). Diffraction data from one crystal were integrated and scaled using XDS, where CC1/2 was used to determine the resolution cutoff^62,63^. Phases were solved using molecular replacement on PHENIX.phaser^64,65^. The molecular replacement model for the variable domain of Fab 1G1 was generated using SWISS model while the constant domain model came from the crystal structure of VRC01 (PDB code: 3NGB)^66^. The molecular replacement model for RSV G CCD came from the crystal structure of RSV G bound to Fab 3D3 (PDB code: 5WNA) using residues 171–186. There was one Fab 1G1-RSV G CCD complex in the asymmetric unit. The electron density map and model were refined using PHENIX.refine while manual assignment and fitting of amino acid side chains into electron density were done using COOT^65,67^. Visualization of final structural features were done in UCSF ChimeraX^68^.

### Production and structure determination of fab 1G8-RSV G CCD complex

A 4-molar excess of recombinant RSV G CCD at 0.3 mg/ml was mixed with Fab 1G8 at 0.5 mg/ml, concentrated to 0.6 mg/ml, dialyzed into 10 mM Tris-Cl pH 8.0 and 150 mM NaCl. This sample was purified on a Superdex75 10/300 SEC column with 50 mM Tris-Cl pH 8.0 and 150 mM NaCl. SEC-purified Fab 1G8-RSV G CCD complex was verified by Coomassie stained SDS-PAGE and was concentrated to 5 mg/ml. Crystals were grown by hanging drop vapor diffusion at 22 °C with well solution containing 0.15 M Sodium Thiocyanate and 16% PEG 3350. Crystals were dipped into a cryoprotectant (well solution containing 25% glycerol) and flash frozen in liquid nitrogen. Frozen crystals were shipped to the Advanced Light Source (ALS) and diffraction data was collected at beamline 5.0.1 using an x-ray wavelength of 0.97Å (12.4 keV). Diffraction data from one crystal were integrated and scaled using DIALS, where CC1/2 was used to determine the resolution cutoff^69–71^. Phases were solved using molecular replacement on PHENIX.phaser^64,65^. The molecular replacement model for the variable domain of Fab 1G8 was generated using SWISS model while the constant domain model came from the crystal structure of VRC01 (PDB code: 3NGB)^66^. The molecular replacement model for RSV G CCD came from the crystal structure of RSV G bound to Fab 3D3 (PDB code: 5WNA) using residues 171–186. There were two Fab 1G8-RSV G CCD complexes in the asymmetric unit. The electron density map and model were refined using PHENIX.refine while manual assignment and fitting of amino acid side chains into electron density were done using COOT^65,67^. Visualization of final structural features were done in UCSF ChimeraX^68^.

### Production and structure determination of fab 2B11-RSV G CCD complex

A 4-molar excess of recombinant RSV G CCD at 0.3 mg/ml was mixed with Fab 2B11 at 0.6 mg/ml, concentrated to 1 mg/ml, dialyzed into 10 mM Tris-Cl pH 8.0 and 150 mM NaCl. This sample was purified on a Superdex75 10/300 SEC column with 50 mM Tris-Cl pH 8.0 and 150 mM NaCl. SEC-purified Fab 2B11-RSV G CCD complex was verified by Coomassie stained SDS-PAGE and was concentrated to 3.1 mg/ml. Crystals were grown by hanging drop vapor diffusion at 22 °C with well solution containing 0.22 M Ammonium Citrate Dibasic and 15% PEG 3350. Crystals were dipped into a cryoprotectant (well solution containing 25% PEG400) and flash frozen in liquid nitrogen. Frozen crystals were shipped to the Advanced Light Source (ALS) and diffraction data was collected at beamline 5.0.1 using an x-ray wavelength of 0.97Å (12.4 keV). Two diffraction datasets from one crystal were integrated and scaled using DIALS where CC1/2 was used to determine the resolution cutoff^69–71^. Phases were solved using molecular replacement on PHENIX.phaser^64,65^. The molecular replacement model for the variable domain of Fab 2B11 was generated using SWISS model while the constant domain model came from the crystal structure of VRC01 (PDB code: 3NGB)^66^. The molecular replacement model for RSV G CCD came from the crystal structure of RSV G bound to Fab 3D3 (PDB code: 5WNA) using residues 171–186. There were seven Fab 2B11-RSV G CCD complexes in the asymmetric unit. The electron density map and model were refined using PHENIX.refine while manual assignment and fitting of amino acid side chains into electron density were done using COOT^65,67^. Visualization of final structural features were done in UCSF ChimeraX^68^.

### Production and cryoem grid preparation of fab 3D3-RSV Gecto-Fab 2D10 complex

Equimolar ratios of size exclusion purified GALNT3 restricted O-glycosylated RSV G^ecto^ and Fab 2D10 were incubated with gentle mixing at 4 °C for one hour before adding a second molar equivalent of Fab 3D3 and a further one hour incubation. The complex was then purified on a Superose 6 increase 10/300 SEC column (Cytiva) in 50 mM Tris-Cl pH 7.5 and 150 mM NaCl and verified on Coomassie stained SDS-PAGE. Purified Fab 3D3-RSV G^ecto^-Fab 2D10 complex was used at 1.4 mg/ml (approximately 10.5 µM). 3 µl of sample was fast incubated with 0.5 µL of octyl-D-glucoside and deposited onto a glow-discharged UltrAuFoil R 2/2 200 gold mesh grid using a ThermoFisher Scientific Vitrobot Mark IV and blotted at 4 °C, 100% humidity for 1.5 s before being plunge-frozen in liquid ethane.

### CryoEM data collection of fab 3D3-RSVGecto-Fab 2D10 complex

Images were collected using an ThermoFisher Scientific Glacios-cryoTwin transmission electron microscope operating at 200 kV using a Gatan K2 Summit direct electron detection and pixel size of 0.69 Å (57,000x) and total dose of 38 e^−^/Å^2^with defocus range between − 0.8 and 3.0 μm^72^. A total of 6,605 movies were collected across all grids and processing using cryoSPARC v4.1^73^. Stage drift and beam-induced anisotropic motion during data collection were corrected using patch-based motion correction^74^. Defocus variation was determined using patch-based CTF estimation. Exposures were manually inspected and curated to remove poor-quality micrographs by setting thresholds for CTF fit resolution (< 10 Å), motion distance, and astigmatism.

### CryoEM data processing

Curated exposures were subjected to automated blob picking using a box size of 40–80 Å resulting in a total of 9,716,771 particles. Picked particles were manually inspected and pruned using a low pass filter, normalized cross-correlation (NCC), and power thresholds to select the highest quality particles. Particles were extracted using a box size of 320 pixels, resulting in a total of 3,159,582 particles and 3,152 movies. Subsequent rounds of 2D classification were performed in order to obtain a suitable set of references for the template picking using a particle diameter of 320 Å, followed by manual inspection of picked particles, particle extraction using a box size of 320 pixels, and 2D classification. Selected 2D classes underwent Ab Initio (67 classes were picked), followed by 3D-classification and the selected set of particles and volume underwent to cycles of non-uniform refinement^75^. The final polishment was performed using local CTF refinement using 50,831 particles. The best 3D reconstructed map was visualized on UCSF-ChimeraX and overlayed crystal structures of Fab 3D3-RSV G CCD (PDB code: 5WNB) and scFv 2D10-RSV G CCD (PDB Code: 5WN9)^68^.

## Electronic supplementary material

Below is the link to the electronic supplementary material.

Supplementary Material 1

## Data Availability

Coordinates and structure factors for the Fab 1G1 - RSV G CCD complex structure, the Fab 1G8 - RSV G CCD complex structure, and the Fab 2B11 - RSV G CCD complex structure have been deposited in the Protein Data Bank (www.rcsb.org) under accession codes 9CQA, 9CQB, and 9CQD, respectively. The electron density map for the Fab 3D3 - RSVGecto - Fab 2D10 complex was deposited in the Electron Microscopy Databank (www.ebi.ac.uk/emdb/) under accession EMD-47153. All data from this study can be requested by email to Rebecca DuBois (rmdubois@ucsc.edu).

## References

[1] Lozano, R. et al. Global and regional mortality from 235 causes of death for 20 age groups in 1990 and 2010: a systematic analysis for the global burden of disease study 2010. *Lancet* **380**, 2095–2128. 10.1016/S0140-6736(12)61728-0 (2012). 23245604 10.1016/S0140-6736(12)61728-0 PMC10790329

[2] Shi, T. et al. Global, regional, and National disease burden estimates of acute lower respiratory infections due to respiratory syncytial virus in young children in 2015: a systematic review and modelling study. *Lancet* **390**, 946–958. 10.1016/S0140-6736(17)30938-8 (2017). 28689664 10.1016/S0140-6736(17)30938-8 PMC5592248

[3] Branche, A. R. & Falsey, A. R. Respiratory syncytial virus infection in older adults: an under-recognized problem. *Drugs Aging*. **32**, 261–269. 10.1007/s40266-015-0258-9 (2015). 25851217 10.1007/s40266-015-0258-9

[4] Falsey, A. R., Hennessey, P. A., Formica, M. A., Cox, C. & Walsh, E. E. Respiratory syncytial virus infection in elderly and high-risk adults. *N Engl. J. Med.* **352**, 1749–1759. 10.1056/NEJMoa043951 (2005). 15858184 10.1056/NEJMoa043951

[5] Falsey, A. R. & Walsh, E. E. Respiratory syncytial virus infection in elderly adults. *Drugs Aging*. **22**, 577–587. 10.2165/00002512-200522070-00004 (2005). 16038573 10.2165/00002512-200522070-00004 PMC7099998

[6] Widmer, K., Griffin, M. R., Zhu, Y., Williams, J. V. & Talbot, H. K. Respiratory syncytial virus- and human metapneumovirus-associated emergency department and hospital burden in adults. *Influenza Other Respir Viruses*. **8**, 347–352. 10.1111/irv.12234 (2014). 24512531 10.1111/irv.12234 PMC3984605

[7] McLaughlin, J. M. et al. Rates of medically attended RSV among US adults: A systematic review and Meta-analysis. *Open. Forum Infect. Dis.* **9**, ofac300. 10.1093/ofid/ofac300 (2022). 35873302 10.1093/ofid/ofac300 PMC9301578

[8] Palivizumab, H. Respiratory syncytial virus monoclonal antibody, reduces hospitalization from respiratory syncytial virus infection in High-risk infants. *Pediatrics* **102**, 531–537 (1998). 9724660

[9] Griffin, M. P. et al. Single-Dose nirsevimab for prevention of RSV in preterm infants. *N Engl. J. Med.* **383**, 415–425. 10.1056/NEJMoa1913556 (2020). 32726528 10.1056/NEJMoa1913556

[10] Topalidou, X., Kalergis, A. M. & Papazisis, G. Respiratory Syncytial Virus Vaccines: A Review of the Candidates and the Approved Vaccines. Pathogens 12. (2023). 10.3390/pathogens12101259 10.3390/pathogens12101259 PMC10609699 37887775

[11] Wilson, E. et al. Efficacy and safety of an mRNA-Based RSV pref vaccine in older adults. *N Engl. J. Med.* **389**, 2233–2244. 10.1056/NEJMoa2307079 (2023). 38091530 10.1056/NEJMoa2307079

[12] Resch, B. Product review on the monoclonal antibody Palivizumab for prevention of respiratory syncytial virus infection. *Hum. Vaccin Immunother*. **13**, 2138–2149. 10.1080/21645515.2017.1337614 (2017). 28605249 10.1080/21645515.2017.1337614 PMC5612471

[13] Olchanski, N. et al. Palivizumab prophylaxis for respiratory syncytial virus: examining the evidence around value. *Open. Forum Infect. Dis.* **5**, ofy031. 10.1093/ofid/ofy031 (2018). 29516023 10.1093/ofid/ofy031 PMC5833316

[14] Suss, R. J. & Simoes, E. A. F. Respiratory syncytial virus Hospital-Based burden of disease in children younger than 5 years, 2015–2022. *JAMA Netw. Open.* **7**, e247125. 10.1001/jamanetworkopen.2024.7125 (2024). 38635270 10.1001/jamanetworkopen.2024.7125 PMC12068875

[15] McMorrow, M. L. et al. Respiratory syncytial Virus-Associated hospitalizations in children < 5 years: 2016–2022. *Pediatrics*10.1542/peds.2023-065623 (2024). 10.1542/peds.2023-065623 PMC11890375 38841769

[16] Walsh, E. E., Peterson, D. R., Kalkanoglu, A. E., Lee, F. E. & Falsey, A. R. Viral shedding and immune responses to respiratory syncytial virus infection in older adults. *J. Infect. Dis.* **207**, 1424–1432. 10.1093/infdis/jit038 (2013). 23382572 10.1093/infdis/jit038 PMC3610422

[17] Battles, M. B. & McLellan, J. S. Respiratory syncytial virus entry and how to block it. *Nat. Rev. Microbiol.* **17**, 233–245. 10.1038/s41579-019-0149-x (2019). 30723301 10.1038/s41579-019-0149-x PMC7096974

[18] McLellan, J. S., Ray, W. C. & Peeples, M. E. Structure and function of respiratory syncytial virus surface glycoproteins. *Curr. Top. Microbiol. Immunol.* **372**, 83–104. 10.1007/978-3-642-38919-1_4 (2013). 24362685 10.1007/978-3-642-38919-1_4 PMC4211642

[19] McLellan, J. S. et al. Structure-based design of a fusion glycoprotein vaccine for respiratory syncytial virus. *Science* **342**, 592–598. 10.1126/science.1243283 (2013). 24179220 10.1126/science.1243283 PMC4461862

[20] McLellan, J. S. et al. Structure of RSV fusion glycoprotein trimer bound to a prefusion-specific neutralizing antibody. *Science* **340**, 1113–1117. 10.1126/science.1234914 (2013). 23618766 10.1126/science.1234914 PMC4459498

[21] Walsh, E. E. et al. Virus-Specific antibody, viral load, and disease severity in respiratory syncytial virus infection. *J. Infect. Dis.* **218**, 208–217. 10.1093/infdis/jiy106 (2018). 29546402 10.1093/infdis/jiy106 PMC6009588

[22] Graham, B. S., Modjarrad, K. & McLellan, J. S. Novel antigens for RSV vaccines. *Curr. Opin. Immunol.* **35**, 30–38. 10.1016/j.coi.2015.04.005 (2015). 26070108 10.1016/j.coi.2015.04.005 PMC4553118

[23] Capella, C. et al. G antibodies, and disease severity in infants and young children with acute respiratory syncytial virus infection. *J. Infect. Dis.* **216**, 1398–1406. 10.1093/infdis/jix489. (2017). 29029312 10.1093/infdis/jix489 PMC5853469

[24] Hause, A. M. et al. Sequence variability of the respiratory syncytial virus (RSV) fusion gene among contemporary and historical genotypes of RSV/A and RSV/B. *PLoS One*. **12**, e0175792. 10.1371/journal.pone.0175792 (2017). 28414749 10.1371/journal.pone.0175792 PMC5393888

[25] Mas, V., Nair, H., Campbell, H., Melero, J. A. & Williams, T. C. Antigenic and sequence variability of the human respiratory syncytial virus F glycoprotein compared to related viruses in a comprehensive dataset. *Vaccine* **36**, 6660–6673. 10.1016/j.vaccine.2018.09.056 (2018). 30292456 10.1016/j.vaccine.2018.09.056 PMC6203811

[26] Simoes, E. A. F. et al. Suptavumab for the prevention of medically attended respiratory syncytial virus infection in preterm infants. *Clin. Infect. Dis.* **73**, e4400–e4408. 10.1093/cid/ciaa951 (2021). 32897368 10.1093/cid/ciaa951 PMC8653633

[27] Langedijk, A. C. et al. A systematic review on global RSV genetic data: identification of knowledge gaps. *Rev. Med. Virol.* **32**, e2284. 10.1002/rmv.2284 (2022). 34543489 10.1002/rmv.2284 PMC9285027

[28] Wilkins, D. et al. Nirsevimab binding-site conservation in respiratory syncytial virus fusion glycoprotein worldwide between 1956 and 2021: an analysis of observational study sequencing data. *Lancet Infect. Dis.* **23**, 856–866. (2023). 10.1016/S1473-3099(23)00062-2 36940703

[29] Zhu, Q. et al. Prevalence and significance of substitutions in the fusion protein of respiratory syncytial virus resulting in neutralization escape from antibody MEDI8897. *J. Infect. Dis.* **218**, 572–580. 10.1093/infdis/jiy189 (2018). 29617879 10.1093/infdis/jiy189

[30] Stray, K. et al. Drug resistance assessment following administration of respiratory syncytial virus (RSV) fusion inhibitor presatovir to participants experimentally infected with RSV. *J. Infect. Dis.* **222**, 1468–1477. 10.1093/infdis/jiaa028 (2020). 31971597 10.1093/infdis/jiaa028

[31] Nziza, N. et al. Longitudinal humoral analysis in RSV-infected infants identifies pre-existing RSV strain-specific G and evolving cross-reactive F antibodies. *Immunity*10.1016/j.immuni.2024.05.019 (2024). 38876099 10.1016/j.immuni.2024.05.019

[32] Johnson, S. M. et al. Respiratory syncytial virus uses CX3CR1 as a receptor on primary human airway epithelial cultures. *PLoS Pathog*. **11**, e1005318. 10.1371/journal.ppat.1005318 (2015). 26658574 10.1371/journal.ppat.1005318 PMC4676609

[33] Jeong, K. I. et al. CX3CR1 is expressed in differentiated human ciliated airway cells and Co-Localizes with respiratory syncytial virus on cilia in a G Protein-Dependent manner. *PLoS One*. **10**, e0130517. 10.1371/journal.pone.0130517 (2015). 26107373 10.1371/journal.pone.0130517 PMC4479564

[34] Chirkova, T. et al. CX3CR1 is an important surface molecule for respiratory syncytial virus infection in human airway epithelial cells. *J. Gen. Virol.* **96**, 2543–2556. 10.1099/vir.0.000218 (2015). 26297201 10.1099/vir.0.000218 PMC4635495

[35] Nziza, N. et al. Longitudinal humoral analysis in RSV-infected infants identifies pre-existing RSV strain-specific G and evolving cross-reactive F antibodies. *Immunity* **57**, 1681–1695e1684. 10.1016/j.immuni.2024.05.019 (2024). 38876099 10.1016/j.immuni.2024.05.019

[36] Jones, H. G. et al. Structural basis for recognition of the central conserved region of RSV G by neutralizing human antibodies. *PLoS Pathog*. **14**, e1006935. 10.1371/journal.ppat.1006935 (2018). 29509814 10.1371/journal.ppat.1006935 PMC5856423

[37] Cortjens, B. et al. Broadly reactive Anti-Respiratory syncytial virus G antibodies from exposed individuals effectively inhibit infection of primary airway epithelial cells. *J. Virol.* **91**10.1128/JVI.02357-16 (2017). 10.1128/JVI.02357-16 PMC5411575 28275185

[38] Caidi, H. et al. Anti-respiratory syncytial virus (RSV) G monoclonal antibodies reduce lung inflammation and viral lung titers when delivered therapeutically in a BALB/c mouse model. *Antiviral Res.* **154**, 149–157. 10.1016/j.antiviral.2018.04.014 (2018). 29678551 10.1016/j.antiviral.2018.04.014 PMC8063470

[39] Boyoglu-Barnum, S. et al. A respiratory syncytial virus (RSV) anti-G protein F(ab’)2 monoclonal antibody suppresses mucous production and breathing effort in RSV rA2-line19F-infected BALB/c mice. *J. Virol.* **87**, 10955–10967. 10.1128/JVI.01164-13 (2013). 23885067 10.1128/JVI.01164-13 PMC3807296

[40] Chirkova, T. et al. Respiratory syncytial virus G protein CX3C motif impairs human airway epithelial and immune cell responses. *J. Virol.* **87**, 13466–13479. 10.1128/JVI.01741-13 (2013). 24089561 10.1128/JVI.01741-13 PMC3838285

[41] Boyoglu-Barnum, S. et al. Prophylaxis with a respiratory syncytial virus (RSV) anti-G protein monoclonal antibody shifts the adaptive immune response to RSV rA2-line19F infection from Th2 to Th1 in BALB/c mice. *J. Virol.* **88**, 10569–10583. 10.1128/JVI.01503-14 (2014). 24990999 10.1128/JVI.01503-14 PMC4178873

[42] Collarini, E. J. et al. Potent high-affinity antibodies for treatment and prophylaxis of respiratory syncytial virus derived from B cells of infected patients. *J. Immunol.* **183**, 6338–6345. 10.4049/jimmunol.0901373 (2009). 19841167 10.4049/jimmunol.0901373

[43] Fedechkin, S. O., George, N. L., Wolff, J. T., Kauvar, L. M. & DuBois, R. M. Structures of respiratory syncytial virus G antigen bound to broadly neutralizing antibodies. *Sci. Immunol.* **3**10.1126/sciimmunol.aar3534 (2018). 10.1126/sciimmunol.aar3534 PMC6203301 29523582

[44] Fedechkin, S. O. et al. Conformational flexibility in respiratory syncytial virus G neutralizing epitopes. *J. Virol.* **94**10.1128/JVI.01879-19 (2020). 10.1128/JVI.01879-19 PMC7158734 31852779

[45] Lee, Y. et al. Monoclonal antibodies targeting sites in respiratory syncytial virus attachment G protein provide protection against RSV-A and RSV-B in mice. *Nat. Commun.* **15**, 2900. 10.1038/s41467-024-47146-2 (2024). 38575575 10.1038/s41467-024-47146-2 PMC10994933

[46] Haynes, L. M. et al. Therapeutic monoclonal antibody treatment targeting respiratory syncytial virus (RSV) G protein mediates viral clearance and reduces the pathogenesis of RSV infection in BALB/c mice. *J. Infect. Dis.* **200**, 439–447. 10.1086/600108 (2009). 19545210 10.1086/600108

[47] Boyoglu-Barnum, S. et al. An anti-G protein monoclonal antibody treats RSV disease more effectively than an anti-F monoclonal antibody in BALB/c mice. *Virology* **483**, 117–125. 10.1016/j.virol.2015.02.035 (2015). 25965801 10.1016/j.virol.2015.02.035 PMC4516680

[48] Tim Beaumont, E. Y. RSV G PROTEIN SPECIFIC ANTIBODIES. United States patent application 15/638.820, June 30, 2017. (2017).

[49] Zhou, T. et al. Structural basis for broad and potent neutralization of HIV-1 by antibody VRC01. *Science* **329**, 811–817. 10.1126/science.1192819 (2010). 20616231 10.1126/science.1192819 PMC2981354

[50] Ekiert, D. C. et al. Cross-neutralization of influenza A viruses mediated by a single antibody loop. *Nature* **489**, 526–532. 10.1038/nature11414 (2012). 22982990 10.1038/nature11414 PMC3538848

[51] Throsby, M. et al. Heterosubtypic neutralizing monoclonal antibodies cross-protective against H5N1 and H1N1 recovered from human IgM + memory B cells. *PLoS One*. **3**, e3942. 10.1371/journal.pone.0003942 (2008). 19079604 10.1371/journal.pone.0003942 PMC2596486

[52] Wu, X. et al. Maturation and diversity of the VRC01-Antibody lineage over 15 years of chronic HIV-1 infection. *Cell* **161**, 470–485. 10.1016/j.cell.2015.03.004 (2015). 25865483 10.1016/j.cell.2015.03.004 PMC4706178

[53] MacCallum, R. M., Martin, A. C. & Thornton, J. M. Antibody-antigen interactions: contact analysis and binding site topography. *J. Mol. Biol.* **262**, 732–745. 10.1006/jmbi.1996.0548 (1996). 8876650 10.1006/jmbi.1996.0548

[54] Camacho, C. et al. BLAST+: architecture and applications. *BMC Bioinform.* **10**, 421. 10.1186/1471-2105-10-421 (2009). 10.1186/1471-2105-10-421 PMC2803857 20003500

[55] Altschul, S. F., Gish, W., Miller, W., Myers, E. W. & Lipman, D. J. Basic local alignment search tool. *J. Mol. Biol.* **215**, 403–410. 10.1016/S0022-2836(05)80360-2 (1990). 2231712 10.1016/S0022-2836(05)80360-2

[56] Sievers, F. et al. Fast, scalable generation of high-quality protein multiple sequence alignments using Clustal Omega. Mol Syst Biol 7, 539. (2011). 10.1038/msb.2011.75 10.1038/msb.2011.75 PMC3261699 21988835

[57] Capella-Gutierrez, S., Silla-Martinez, J. M. & Gabaldon, T. TrimAl: a tool for automated alignment trimming in large-scale phylogenetic analyses. *Bioinformatics* **25**, 1972–1973. 10.1093/bioinformatics/btp348 (2009). 19505945 10.1093/bioinformatics/btp348 PMC2712344

[58] Tareen, A. & Kinney, J. B. Logomaker: beautiful sequence logos in Python. *Bioinformatics* **36**, 2272–2274. 10.1093/bioinformatics/btz921 (2020). 31821414 10.1093/bioinformatics/btz921 PMC7141850

[59] Fairhead, M. & Howarth, M. Site-specific biotinylation of purified proteins using BirA. *Methods Mol. Biol.* **1266**, 171–184. 10.1007/978-1-4939-2272-7_12 (2015). 25560075 10.1007/978-1-4939-2272-7_12 PMC4304673

[60] Yang, Z. et al. The GalNAc-type O-Glycoproteome of CHO cells characterized by the simplecell strategy. *Mol. Cell. Proteom.* **13**, 3224–3235. 10.1074/mcp.M114.041541 (2014). 10.1074/mcp.M114.041541 PMC4256479 25092905

[61] Nagashima, K. & Mousa, J. J. Epitope Binning of monoclonal and polyclonal antibodies by biolayer interferometry. *Methods Mol. Biol.* **2673**, 17–32. 10.1007/978-1-0716-3239-0_2 (2023). 37258904 10.1007/978-1-0716-3239-0_2

[62] Evans, P. R. & Murshudov, G. N. How good are my data and what is the resolution? *Acta Crystallogr. D Biol. Crystallogr.* **69**, 1204–1214. 10.1107/S0907444913000061 (2013). 23793146 10.1107/S0907444913000061 PMC3689523

[63] Agirre, J. et al. The CCP4 suite: integrative software for macromolecular crystallography. *Acta Crystallogr. D Struct. Biol.* **79**, 449–461. 10.1107/S2059798323003595 (2023). 37259835 10.1107/S2059798323003595 PMC10233625

[64] McCoy, A. J. et al. Phaser crystallographic software. *J. Appl. Crystallogr.* **40**, 658–674. 10.1107/S0021889807021206 (2007). 19461840 10.1107/S0021889807021206 PMC2483472

[65] Liebschner, D. et al. Macromolecular structure determination using X-rays, neutrons and electrons: recent developments in phenix. *Acta Crystallogr. D Struct. Biol.* **75**, 861–877. 10.1107/S2059798319011471 (2019). 31588918 10.1107/S2059798319011471 PMC6778852

[66] Waterhouse, A. et al. SWISS-MODEL: homology modelling of protein structures and complexes. *Nucleic Acids Res.* **46**, W296–W303. 10.1093/nar/gky427 (2018). 29788355 10.1093/nar/gky427 PMC6030848

[67] Emsley, P., Lohkamp, B., Scott, W. G. & Cowtan, K. Features and development of Coot. *Acta Crystallogr. D Biol. Crystallogr.* **66**, 486–501. 10.1107/S0907444910007493 (2010). 20383002 10.1107/S0907444910007493 PMC2852313

[68] Meng, E. C. et al. UCSF chimerax: tools for structure Building and analysis. *Protein Sci.* **32**, e4792. 10.1002/pro.4792 (2023). 37774136 10.1002/pro.4792 PMC10588335

[69] Potterton, L. et al. CCP4i2: the new graphical user interface to the CCP4 program suite. *Acta Crystallogr. D Struct. Biol.* **74**, 68–84. 10.1107/S2059798317016035 (2018). 29533233 10.1107/S2059798317016035 PMC5947771

[70] Winter, G. et al. DIALS: implementation and evaluation of a new integration package. *Acta Crystallogr. D Struct. Biol.* **74**, 85–97. 10.1107/S2059798317017235 (2018). 29533234 10.1107/S2059798317017235 PMC5947772

[71] Beilsten-Edmands, J. et al. Scaling diffraction data in the DIALS software package: algorithms and new approaches for multi-crystal scaling. *Acta Crystallogr. D Struct. Biol.* **76**, 385–399. 10.1107/S2059798320003198 (2020). 32254063 10.1107/S2059798320003198 PMC7137103

[72] Mastronarde, D. N. SerialEM: A program for automated Tilt series acquisition on Tecnai microscopes using prediction of specimen position. *Microsc. Microanal.* **9**10.1017/S1431927603445911 (2003).

[73] Punjani, A., Rubinstein, J. L., Fleet, D. J. & Brubaker, M. A. CryoSPARC: algorithms for rapid unsupervised cryo-EM structure determination. *Nat. Methods*. **14**, 290–296. 10.1038/nmeth.4169 (2017). 28165473 10.1038/nmeth.4169

[74] Rubinstein, J. L. & Brubaker, M. A. Alignment of cryo-EM movies of individual particles by optimization of image translations. *J. Struct. Biol.* **192**, 188–195. 10.1016/j.jsb.2015.08.007 (2015). 26296328 10.1016/j.jsb.2015.08.007

[75] Punjani, A., Zhang, H. & Fleet, D. J. Non-uniform refinement: adaptive regularization improves single-particle cryo-EM reconstruction. *Nat. Methods*. **17**, 1214–1221. 10.1038/s41592-020-00990-8 (2020). 33257830 10.1038/s41592-020-00990-8

[76] Croll, T. I. ISOLDE: a physically realistic environment for model Building into low-resolution electron-density maps. *Acta Crystallogr. D Struct. Biol.* **74**, 519–530. 10.1107/S2059798318002425 (2018). 29872003 10.1107/S2059798318002425 PMC6096486

