# Supplementary material for: Structures of respiratory syncytial virus G bound to broadly reactive antibodies provide insights into vaccine design
Source: Sci Rep. 2025 Mar 13;15:8666. doi: 10.1038/s41598-025-92886-w (PMC11906780; doi:10.1038/s41598-025-92886-w)
Supplement: Supplementary file 1 — Supplementary Material 1 [file 41598_2025_92886_MOESM1_ESM.pdf]

Supplementary Information

Structures of respiratory syncytial virus G bound to broadly-reactive antibodies provide insights into vaccine design

Maria G. Juarez[a], Sara M. O'Rourke[b,] John V. Dzimianski[b], Delia Gagnon[b], Gabriel Penunuri[b,c], Vitor H. B. Serrão[d,e], Russell B. Corbett-Detig[b,c], Lawrence M. Kauvar[f], and Rebecca M. DuBois[b#]

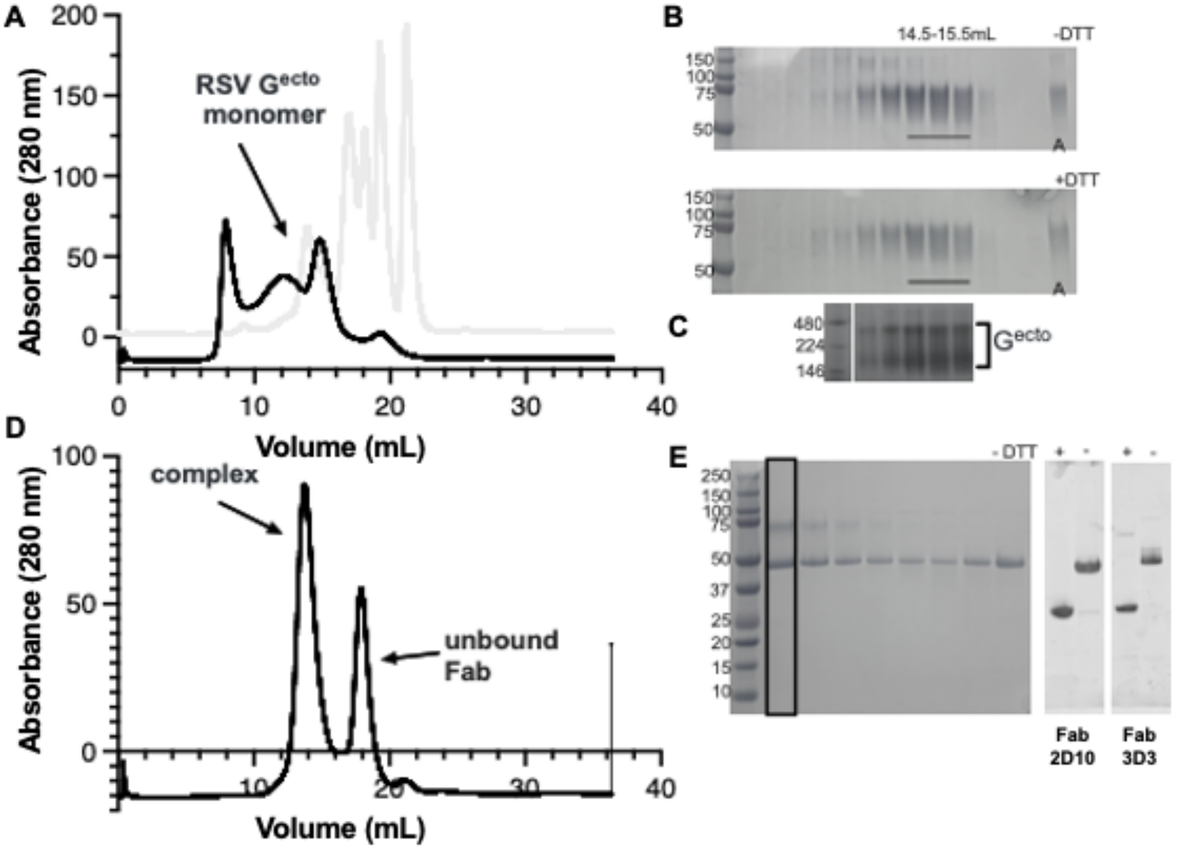

Supplementary Figure 1. (A) Affinity-purified RSV G^{ecto} produced in a SimpleCell (Cosmc-KO) GALNT3+ CHO K1 cells was polished to remove aggregates by size exclusion chromatography on a Superose 6 increase 10/300 column in 50 mM Tris-Cl pH 7.5 and 300 mM NaCl. (B) Monomeric RSV G^{ecto} was identified by denaturing electrophoresis of eluted protein, in the presence and absence of a reducing agent. (C) Novex Blue Native Page gel (Thermo-Fisher) of fractions 14.5-15.5 mL. Two distinct bands were identified; the higher MW moiety may represent protein decorated with complex charged N-glycans, and the lower band protein with less complex high-mannose type glycans. Unglycosylated protein has a predicted MW of ~30kDa. (D) Fab 3D3-RSV G^{ecto}-Fab 2D10 complex was purified on a Superose 6 increase 10/300 column in 50 mM Tris-Cl pH 7.5 and 150 mM NaCl, verified on a denaturing Coomassie stained SDS-PAGE gel (E).

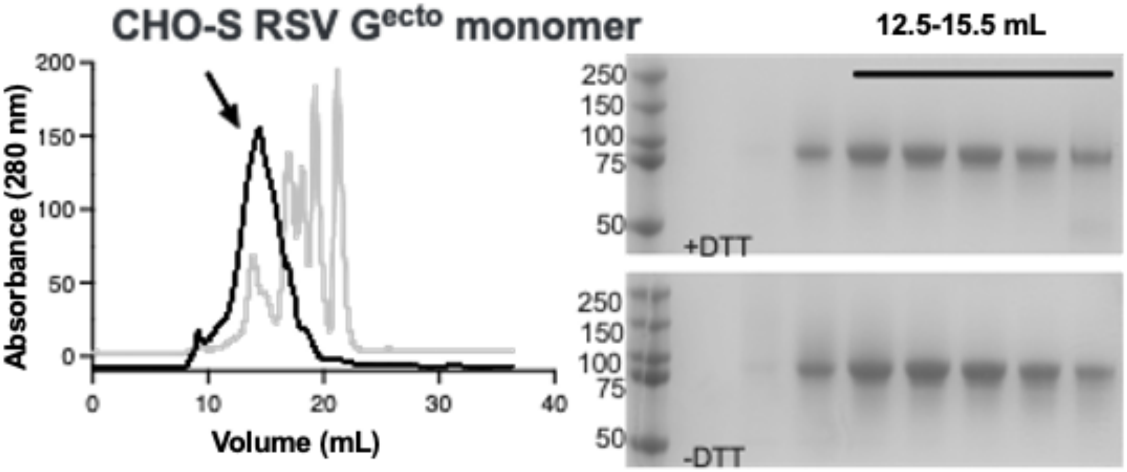

Supplementary Figure 2. Affinity purified RSV G^{ecto} produced in CHO-S cells was polished on a Superose 6 increase 10/300 SEC column in 50 mM Tris-Cl pH 7.5 and 300 mM NaCl and verified on Coomassie stained SDS-PAGE gels.

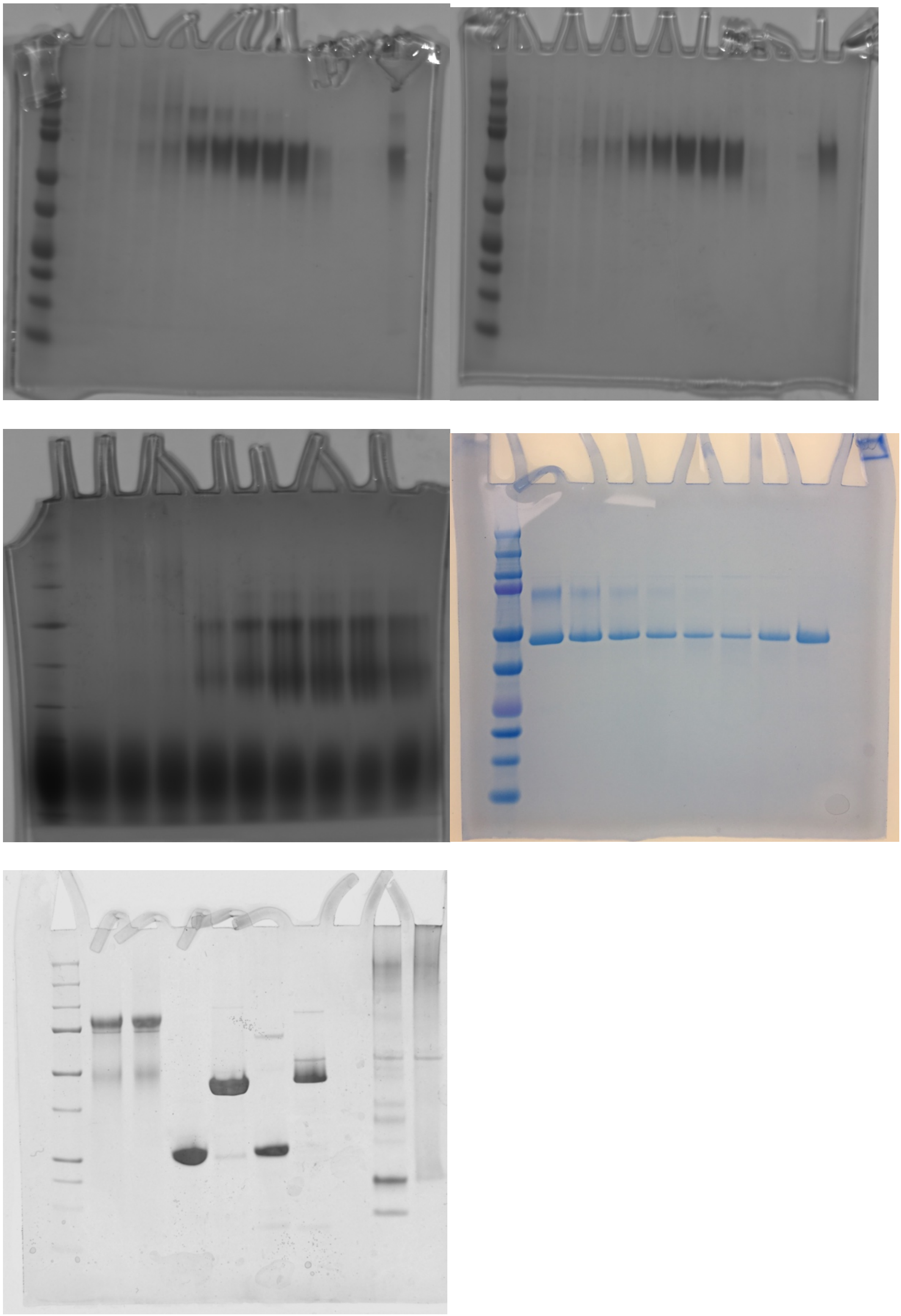

Supplementary Figure 3. Uncropped Coomassie stained SDS-PAGE gels from Supplementary Figure 1.

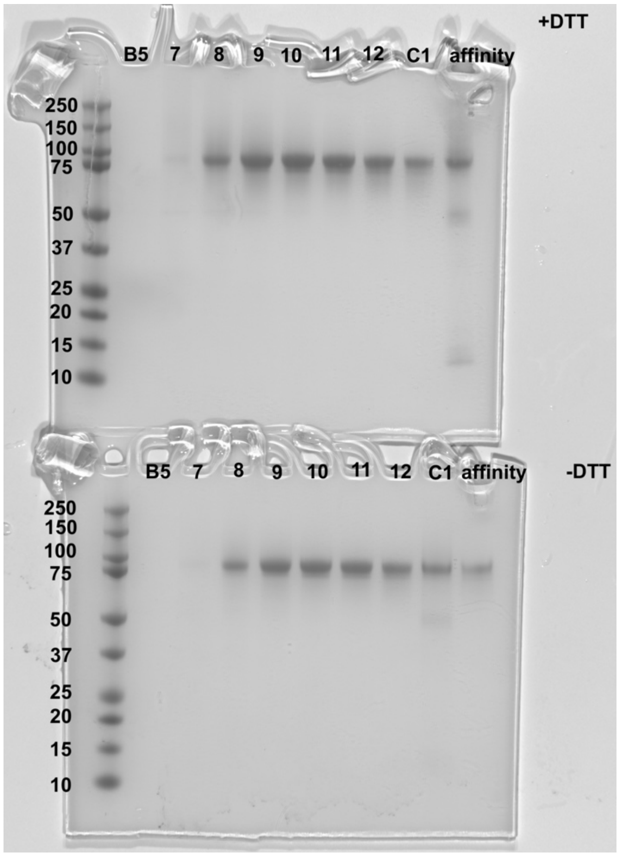

Supplementary Figure 4. Uncropped Coomassie stained SDS-PAGE gels from Supplementary Figure 2.
